# Chemoenzymatic multistep retrosynthesis with transformer loops†

**DOI:** 10.1039/d4sc02408g

**Published:** 2024-10-08

**Authors:** David Kreutter, Jean-Louis Reymond

**Affiliations:** a Department of Chemistry, Biochemistry and Pharmaceutical Sciences, University of Bern Freiestrasse 3 3012 Bern Switzerland david.kreutter@unibe.ch jean-louis.reymond@unibe.ch

## Abstract

Integrating enzymatic reactions into computer-aided synthesis planning (CASP) should help devise more selective, economical, and greener synthetic routes. Herein we report the triple-transformer loop algorithm with biocatalysis (TTLAB) as a new CASP tool for chemo-enzymatic multistep retrosynthesis. Single-step retrosyntheses are performed using two triple transformer loops (TTL), one trained with chemical reactions from the US Patent Office (USPTO-TTL), the second one obtained by multitask transfer learning combining the USPTO dataset with preparative biotransformations from the literature (ENZR-TTL). Each TTL performs single-step retrosynthesis independently by tagging potential reactive sites in the product, predicting for each site possible starting materials (T1) and reagents or enzymes (T2), and validating the predictions *via* a forward transformer (T3). TTLAB combines predictions from both TTLs to explore multistep sequences using a heuristic best-first tree search and propose short routes from commercial building blocks including enantioselective biocatalytic steps. TTLAB can be used to assist chemoenzymatic route design.

## Abstract 2

Integrating enzymatic reactions into computer-aided synthesis planning (CASP) should help devise more selective, economical, and greener synthetic routes.

## Graphical abstract

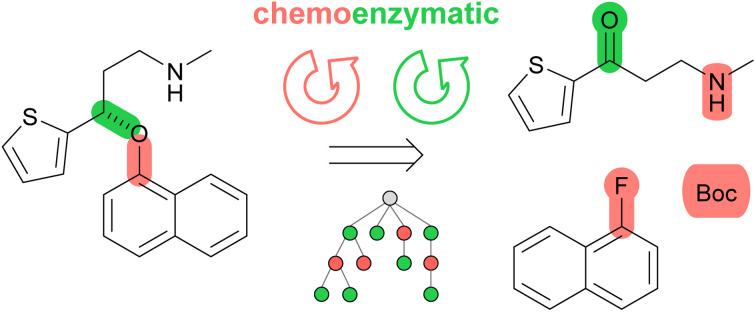

## Introduction

Computer-aided synthesis planning (CASP), originally proposed by E. J. Corey in the 1960's, uses computational approaches, including rule-based systems as well as various types of neural networks, to exploit synthetic methodology as recorded in the scientific literature to propose multistep syntheses of target molecules from commercial precursors.^1–27^ Integrating enzyme-catalyzed reactions would enable CASP to participate in the global effort towards more selective, economical, and greener chemical manufacturing processes. However, the task is challenging due to the sparsity and very different nature of biotransformations compared to chemical reactions.^28–33^ Both template-based and transformer-based CASP tools for biocatalysis were recently reported,^34–36^ which make use of biochemical reaction data describing mostly metabolic pathways as collected in databases such as BRENDA, KEGG, MetaCyc, Rhea, PathBank, MetaNetX or EzCatDB.^37–43^ However, these biochemical pathway datasets only partly reflect the use of enzymes in organic synthesis, where enzymes or enzyme preparations (extracts, whole cells, *etc.*) are used under non-natural conditions, such as in immobilized form and at very high substrate concentrations, and to convert molecules often quite different from the natural substrate.^31^ The CASP tool ASKCOS,^44,45^ on the other hand, proposes chemo-enzymatic route finding using a template-based strategy based on literature data collected from the Reaxys database^46^ for both chemistry and biocatalysis, which for the case of enzymes represent more relevant examples for the practice of organic synthesis compared to data from biochemical pathways.

We recently showed that CASP tools based on transformer models,^17,18^ trained on SMILES descriptions^47,48^ of chemical reactions of starting materials (SM) with a set of reagents (R) to form a product (P) as collected in the public USPTO dataset,^49,50^ can be adapted to specific reaction subclasses by transfer learning.^51^ Extending on this opportunity, we then showed that literature information on a few ten thousand biotransformations extracted from Reaxys,^46^ for which the reagent set R is substituted with a text description of the enzyme or enzyme preparation, can be combined with the USPTO dataset to train a transformer model by multi-task transfer learning (MTL).^52^ The resulting enzymatic transformer performed forward predictions of enzymatic reactions as used in typical preparative biotransformations, including enantioselective processes such as kinetic resolution with lipases or enantioselective ketone reduction and reductive aminations with 71% top-2 accuracy, approaching the typical performance of forward transformer models. The key difference between our enzymatic transformer model and the other approaches for enzymatic reactions mentioned above was the use of a text description of the enzyme rather than its E.C classification or a link to literature references.

**Fig. 1:**
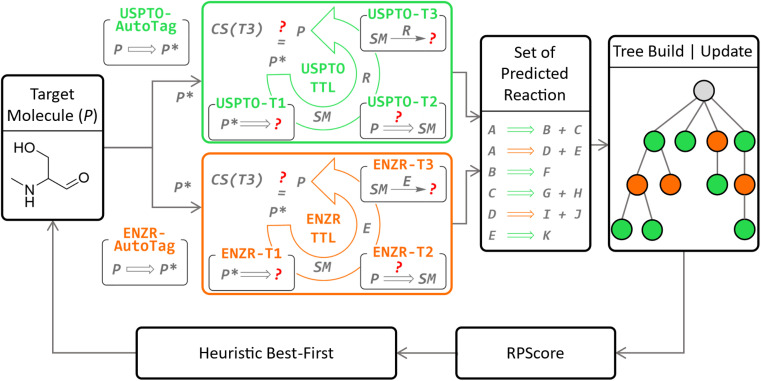
Concept of the TTLAB multistep search operating organic (USPTO-TTL, green panel)^53^ and enzymatic (ENZR-TTL, orange panel) catalysis in parallel. In the new enzymatic retrosynthesis, potential reactive sites in a product molecule P are first labelled by the new model ENZR-Autotag, and each labelled product P* is then passed through ENZR-TTL consisting of the new models ENZR-T1 predicting starting materials (SM) from P*, ENZR-T2 predicting the enzyme name E from SM → P, and the previously reported forward model ENZR-T3 predicting P from SM + E. The retrosynthetic step is validated if the correct product P is predicted by ENZR-T3 with a confidence score CS(T3) above 95%. The confidence scores CS(T3) are used to compute the RPScore^53^ to prioritize steps in the retrosynthetic tree *via* a heuristic best-first sorting.

Herein we report the integration of our enzymatic transformer model into our recently reported triple transformer loop algorithm (TTLA) for multistep chemical retrosynthesis,^53^ to obtain a triple transformer loop algorithm with biocatalysis (TTLAB, Fig. 1). Our previously reported triple transformer loop algorithm (TTLA) performed single-step retrosynthesis prediction using a triple transformer loop (TTL) operating on products P with tagged reactive sites to explore diverse bond disconnections. In detail, potential reactive sites in P were first tagged to produce a series of P*,^54^ and for each P* a first transformer T1 was used to predict SM, a second transformer T2 to predict a suitable R for the proposed transformation SM → P, and finally a third transformer T3 to predict P from the predicted SM and R, thereby potentially validating the retrosynthetic step. The TTLAB presented here combines our original triple transformer loop trained on USPTO,^53^ here called USPTO-TTL, with ENZR-TTL, which is a new TTL trained on an updated version of our previously reported ENZR dataset containing biotransformations from the literature and originally used only for forward predictions,^52^ which now comprises 57 176 reactions.

To predict enzymatic disconnections, ENZR-TTL tags potential reactive sites in P to produce various tagged P* by using a new tagging model for enzymatic disconnections trained on ENZR, called ENZR-AutoTag. ENZR-TTL then predicts possible SM from each possible tagged P* using a new ENZR-T1 model, and possible enzymes (E) for the predicted reaction SM → P by a new ENZR-T2 model. Both transformer models are obtained by MTL of the USPTO dataset with the ENZR dataset. Finally, ENZR-TTL validates the predicted SM + E → P reaction with the previously reported forward transformer ENZR-T3 (retrained on a more recent and larger ENZR dataset) based on the identity of the predicted and original P and the confidence score.^52^

To explore multistep chemo-enzymatic retrosyntheses, TTLAB considers single-step predictions from both USPTO-TTL and ENZR-TTL using the approach developed previously for TTLA. In this approach, possible routes are ranked with the route penalty score (RPScore),^53^ combining the simplicity of all SM along the route,^55,56^ with the confidence score of each retrosynthetic step, as well as route length, and the various routes are ranked and iteratively extended using a heuristic best-first tree search. TTLAB can be used to assist chemoenzymatic route design.

## Methods

### Chemical reaction dataset

The same United States Patent and Trademark Office (USPTO) chemical reaction dataset as in our previous report was used.^53^ It is a version curated by Thakkar *et al.*^54^ derived from the data mining work of Lowe.^49,50^

### Triple transformer loop models for chemical reactions (USPTO-TTL)

The models trained on the USPTO dataset are identical as in our previous study and available on Zenodo,^53^ and herein named USPTO-TTL. AutoTag is a tagging model predicting tagged product P* from the target product P. T1 is a disconnection-aware retrosynthesis model predicting starting materials SM from the target tagged product P*. T2 is a reaction condition model predicting reagents R, including catalyst and solvent, from the reaction SM → P. T3 is a forward validation model predicting P from SM + R.^57^

### Enzymatic dataset

The enzymatic reaction dataset, herein named ENZR, was extracted from Reaxys using the API accessible under a commercial license.^46^ We first isolated reactions labelled as “enzymatic reaction” in the “other conditions” field (“RXD.COND”). Next, we compiled a list of reagents, catalysts, and solvents typically associated with enzymatic reactions. This involved identifying components with the “ase” suffix in the text fields “RXD.RGT,” “RXD.CAT,” and “RXD.SOL,”. Additionally, we manually selected keywords corresponding to enzymatic transformations, such as “P450,” “NADP,” “CAL-B,” “flavin mononucleotide,” and others, from the most frequently occurring reagents and catalysts in the initial data retrieval. Finally, we queried these enzymatic components individually in the Reaxys database and retrieved the associated reactions. This process resulted in a raw dataset consisting of 107 865 enzymatic reactions.

### Enzymatic dataset: cleaning

The process of cleaning the ENZR dataset involved several steps, wherein the RDKit library was used across various stages.^58^ Initially, multistep reactions and those lacking any reactant or product were excluded, leaving 95 389 reactions. Next, reactions were mapped using RxnMapper,^59^ for which 1333 reactions failed and were removed. Reactions with unspecified atomic symbols (“*”) were also removed. Unmapped reactant molecules were removed for each reaction. A significant number of reactions (32 527) with more than one product were removed. The remaining reactions were tagged with reactive atoms as described previously,^53^ and reactions with no tagged atoms, or with more than 10 tagged atoms, were removed. This cleaning process results in a final enzymatic dataset of 57 176 unique reactions SMILES^47,48^ associated with textual descriptions of each reagent, including cofactors, enzymes, and solvent.

### Enzymatic AutoTag and triple transformer loop (ENZR-TTL) models

Enzymatic transformer models for the ENZR-TTL, including the AutoTag to tag reactive sites, and T1, T2 and T3 in the TTL itself, were trained using the USPTO and the ENZR dataset through MTL, similar to our previous Enzymatic Transformer model with identical training hyperparameters.^52^ The split ratio 90 : 5 : 5 was applied as in the USPTO dataset resulting in 51 459 : 2859 : 2858 reactions in the training, validation, and test set respectively. The dataset split was done such that reactions resulting in identical products belong to the same splitting set.

During the MTL processes detailed below for all ENZR models, we incorporated instruction tokens. These tokens, “ENZYME” for the ENZR dataset and “USPTO” for the USPTO dataset, were inserted at both the start and end of the SMILES inputs. This addition aimed to provide additional context to the model and enable it to focus on specific prediction types as needed.

The ENZR-AutoTag model was trained to predict the tagged SMILES of the product (P*) from the product SMILES (P), in a similar manner to the USPTO-AutoTag model. The ENZR-T1 was trained to predict SM from P* for enzymatic retrosynthesis. In contrast, the ENZR-T2 model differs significantly from its USPTO-T2 counterpart by predicting a textual description of the enzyme (TDE) rather than reagents (R) in SMILES format from the theoretical reaction SMILES (SM → P). The ENZR-T3, previously reported as the Enzymatic Transformer,^52^ serves as forward validation, it was trained from SM + TDE to predict P, now retrained using the new ENZR dataset.

### Disconnection-aware automatic tagging strategy

In our previous study,^53^ the USPTO-TTL employed a combination of three tagging strategies: (1) a systematic tagging procedure, tagging 1 to 3 neighbouring atoms, (2) tagging templates of reactive sites with a conditional structure radius of 2 atoms, and (3) the AutoTag Transformer model with a beam size of 50.

The ENZR-TTL uses a specific tagging strategy combining only an AutoTag model^54^ and templates, excluding the systematic tagging approach. The dedicated ENZR-AutoTag was trained from the ENZR dataset and USPTO by MTL. ENZR reactive site templates were extracted from ENZR exclusively with a radius of 2 atoms.

### Chemoenzymatic multistep tree search algorithm

In parallel to the existing single-step USPTO-TTL, we added the ENZR-TTL, which the multistep algorithm uses systematically and independently. The prediction outcomes of both TTLs are provided to the heuristic best-first tree search, elaborating routes mixing the predictions of both TTLs. Confidence scores of both TTLs behaving differently, the confidence scores of ENZR-T3 were adapted by polynomial fit to the USPTO-T3 distribution (Fig. S1†) to ensure a fair scoring across TTLs. The RPScore, based on molecular simplicity^55,56^ and confidence scores of T3 distinguishes which routes are the best to explore further, and functions the same as reported in our previous study.^53^

Our previous report of the Enzymatic Transformer model, herein named ENZR-T3, demonstrated that a confidence score threshold was required to filter unreasonable enzymatic reactions. A similar evaluation using the round-trip evaluation of the ENZR-TTL was performed and a threshold of 90% confidence of ENZR-T3 was defined for considering ENZR-TTL predictions for multistep retrosynthesis search.

### Building block (BB) set

We combined MolPort (https://www.molport.com) and Enamine (https://www.enamine.net) databases to build a database of 534 058 commercially available compounds as the building block (BB) set.

## Results and discussion

Realizing the triple transformer loop algorithm with biocatalysis (TTLAB) for chemoenzymatic retrosynthesis required first to select a suitable dataset of enzymatic reactions, second to adapt our previous chemical reaction TTL to these enzymatic reactions, and finally to combine the enzymatic reaction TTL with the chemical reaction TTL in a multistep search algorithm. These steps are described in the following subsections.

### Chemical and enzymatic reaction datasets and their comparison

We used the USPTO reaction dataset, which lists one million chemical reactions taken from the patent literature, as a broadly accepted selection of chemical reactions used in organic synthesis.^49,50^ In terms of enzymatic reactions, we selected 57 176 enzymatic reactions from the scientific literature using the Reaxys API,^46^ forming an enlarged version of our earlier enzymatic reaction dataset (ENZR, see methods for details).^52^ The composition of this enlarged ENZR dataset is comparable to its smaller version and reflects the practice of biocatalysis in preparative organic chemistry as reported in the scientific literature, with lipases and dehydrogenases forming the largest class of enzymes (Fig. S2†).

**Fig. 2:**
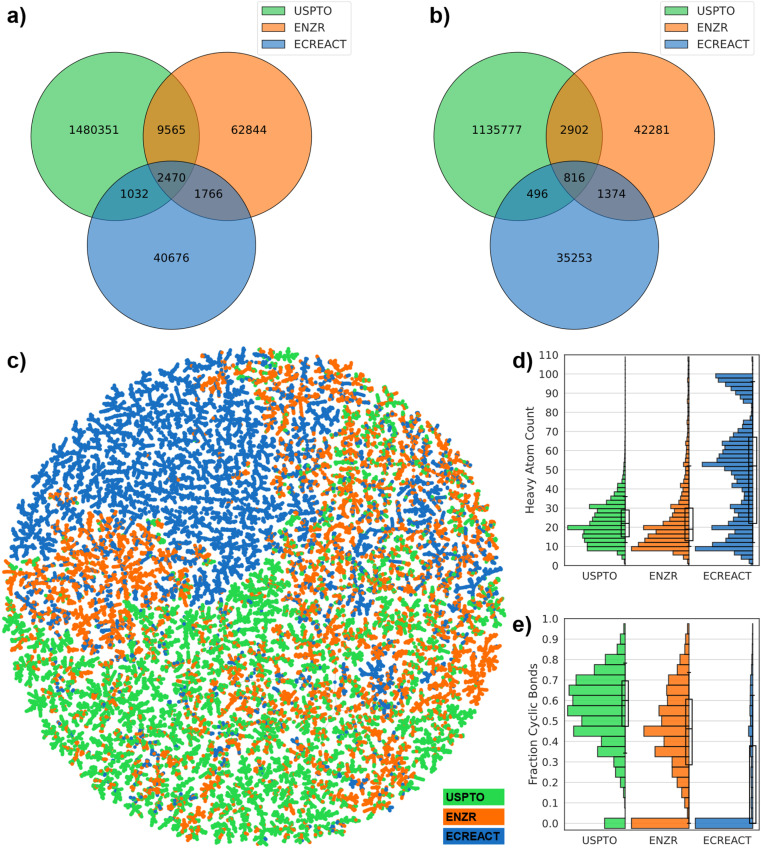
Comparative analysis of USPTO, ENRZ and ECREACT datasets. (a) Venn diagram of all molecules in the USPTO, ENZR and the ECREACT datasets. (b) Venn diagram for only products (P) of reactions. (c) TMAP of 3 × 10 000 randomly chosen molecules from USPTO, ENZR and ECREACT datasets with similarities computed with the MAP4 fingerprint. The interactive map is available at https://tm.gdb.tools/TTLA/EnzymeDB.html. (d) Number of heavy atoms distribution for molecules in each dataset. (e) Fraction of cyclic bond distribution for molecules in each dataset.

In view of training transformer models for a combined chemoenzymatic retrosynthesis, we analyzed whether the 57 176 enzyme-catalyzed reactions in our ENZR dataset contained starting materials and products comparable to those in USPTO. We also analyzed the ECREACT data,^36^ which lists 62 222 enzyme-catalyzed reactions associated with their respective enzyme commission (EC) number, aggregated from the biochemical reaction pathways datasets Rhea, BRENDA, PathBank, and MetaNetX (Table 1).^37,41–43^ ENZR listed fewer reactions than ECREACT but more molecules, indicating a larger diversity of molecules tested in preparative biocatalysis compared to biochemical intermediates. Furthermore, ENZR shared a larger number of molecules with USPTO than ECREACT, and only shared a small number of molecules with ECREACT. A similar distribution was observed when focusing only on reaction products, with only 2470 molecules and 816 product molecules being shared between all three datasets (Fig. 2a and b).

**Table 1:** Dataset information

	USPTO	ENZR	ECREACT
Number of reactions	1 266 734	57 176	62 222
Number of unique molecules	1 493 418	76 645	45 944
Number (%) of molecules shared with USPTO	—	12 035 (15.7%)	3502 (7.6%)
Number (%) of molecules shared with ECREACT	3502 (0.27%)	4236 (7.4%)	—
Number of chiral molecules	271 504	45 277	34 177

To compare the three datasets in terms of molecule types, we selected 10 000 molecules randomly across starting materials and products in each dataset and constructed a TMAP,^60^ employing the MinHashed atom-pair fingerprint MAP4 as similarity measure, which considers substructures and their relative position in molecules.^61^ Areas of the TMAP covered by molecules from USPTO (green) also contained molecules from ENZR (orange), and to a lesser extent from ECREACT (blue), showing a certain level of overlap in structural types between the three datasets (Fig. 2c). Nevertheless, parts of the map were dominated by one of three datasets. Predominantly green areas (USPTO) contained drug-like heteroaromatic molecules, while predominantly orange areas (ENZR) featured glycosides and peptides. Furthermore, one fourth of the TMAP was standing out because it was entirely blue (ECREACT) and was populated by phospholipids and triglycerides apparently completely absent from the other two datasets, probably reflecting the difficulty to work with such molecules in terms of preparative organic synthesis.

Histograms further highlighted similarities and differences between molecules composing the three datasets. A histogram of molecular size as heavy atom count (HAC) showed that ENZR and USPTO contained molecules of comparable size (10 ≤ HAC ≤ 40), while more than half of ECREACT contained larger molecules (HAC > 40) (Fig. 2d). Furthermore, a histogram of the fraction of cyclic bonds showed that USPTO contained mostly cyclic molecules, while ENZR contained similarly cyclic molecules but also a sizable fraction of entirely acyclic molecules, and ECREACT was almost entirely composed of acyclic molecules (Fig. 2e). The difference in molecule properties between the three datasets was also visible in scatter plots using molecular weight, the fraction of carbon atoms and the fraction of cyclic bonds as molecular descriptors (Fig. S3†). Note that 47.9% of ECREACT molecules contained a phosphate functional group, compared to 8.2% in ENZR molecules and only 0.5% in USPTO molecules, further highlighting the different nature of molecules involved in biochemical reaction pathways compared to those in use for synthetic chemistry.

Taken together, these comparisons showed that molecules in ENZR and USPTO datasets showed a significant level of overlap and might be useful for a transformer model approach for combined chemoenzymatic retrosynthesis. By contrast, the differences between ECREACT and USPTO were more pronounced and suggested that these two datasets were almost incompatible with each other.

### Enzymatic triple transformer loop (ENZR-TTL)

Our TTL approach for single-step retrosynthesis consists of tagging potential reactive sites in the product molecule P to form a series of tagged P*, and for each P* to apply three subsequent transformer models predicting SM from P* (T1), reagents R from SM → P (T2), and finally product P from SM + R (T3). T3 validates the retrosynthetic step if the predicted P is identical to the input P, and the confidence score of the T3 prediction is used to compute the route penalty score (RPScore) for the multistep search.^53^

In our approach, potential reactive sites in the product molecule are first tagged to mark potential reactive sites. Our chemical reaction TTL used a combination of a transformer model, templates and systematic tagging. Due to the much higher substrate specificity of enzymes compared to chemical reagents, we removed the systematic tagging approach for our enzymatic TTL and only considered tagging with a transformer model and with templates. Reactive sites in product molecules of the ENZR dataset were identified from atom-mapping and labelled as previously described for the USPTO.^53^ An ENZR-AutoTag transformer was then trained by MTL combining the tagged and untagged datasets of ENZR and USPTO. Enzymatic templates were extracted from the atom-mapped ENZR dataset considering only templates with a radius of two bonds around reacting atoms to take enzyme specificity into account, an aspect which was also reflected by the much smaller number of ENZR templates (18 083) compared to the number of USPTO templates (281 153).

To complement the transformer models for the chemical TTL trained with the USPTO dataset (here named USPTO-TTL), we used MTL of USPTO with the ENZR dataset using the previously described parameters^52^ to obtain models for the enzymatic TTL (here named ENZR-TTL). To help the transformers to learn the differences between chemical and enzymatic reactions, all entries for MTL were labelled before and after the SMILES with “ENZYME” for ENZR data, and with “USPTO” for USPTO data. These labels helped to avoid task ambiguity between USPTO *vs.* ENZR caused by the substitution of reagent SMILES with enzyme names in text format for T2 (SMILES → SMILES *vs.* SMILES → text) and T3 (SMILES → SMILES *vs.* SMILES + text → SMILES). The influence of the instruction tokens “ENZYME” and “USPTO” added before and after each input was well visible in the case of ENZR-T2, for which the fraction of textual enzyme description produced increased from 85.3% for an uninstructed model to 99.7% for the instructed model.

**Fig. 3:**
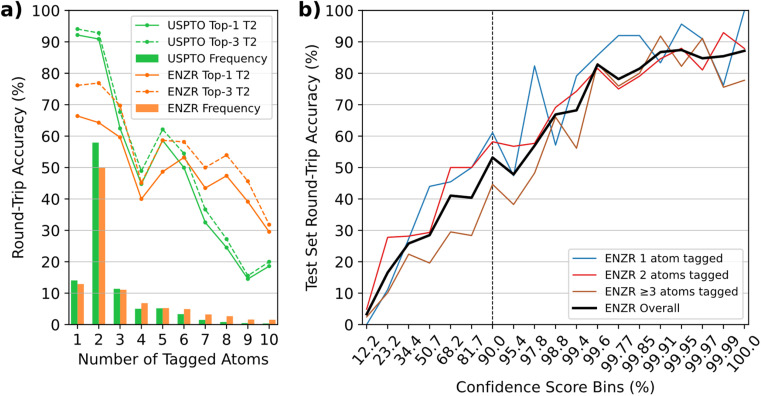
(a) Round-trip accuracies of ENZR-TTL and USPTO-TTL as function of the number of tagged atoms on the target molecules from the ENZR and USPTO test sets respectively. The top-N represents the round-trip accuracy considering multiple examples of enzyme textual descriptions predicted by ENZR-T2 or reagents predicted by USPTO-T2. The bar plots show the frequency fractions as function of the number of tagged atoms for both test sets. (b) Round-trip accuracy of ENZR-TTL as function of confidence scores of ENZR-T3. The vertical dashed bar represents the chosen confidence score cut-off. Bins were selected to equally distribute predictions.

In terms of single-step round-trip accuracy,^57^ the ENZR-TTL achieved 59.0% top-1 accuracy on the ENZR test set, somewhat below the 81.3% top-1 accuracy of the USPTO-TTL on the USPTO test set. In both cases, the top-1 round-trip accuracy measured the percentage of cases where P predicted by T3 matched the input P, which also included cases with different SM and R compared to the ground truth in the test sets (see details in Tables S1 and S2†). In both TTLs, the round-trip accuracy decreased as function of the increasing number of tagged atoms, suggesting that the decreasing number of training examples and the increasing reaction complexity caused more difficult learning in the different transformers involved in producing the TTL predictions (Fig. 3a). ENZR-TTL top-3 round-trip accuracies were as high as 76.2% and 76.9% for single and double atom tags, compared to 94.1% and 92.8% in the case of USPTO-TTL. The lower performance of ENZR-TTL compared to USPTO-TTL probably reflects the smaller training set of enzymatic reactions learned by transfer learning, and a more difficult task associated with the prediction of enzyme names in T2. A similar analysis on the round-trip accuracy as function of the heavy atom count showed no significant higher accuracies on smaller molecules, but rather a dependence on the number of molecules per molecular size bin, emphasizing again a dependence on training set size rather than on molecular size (Fig. S4†). As for the USPTO-T3, the confidence score of ENZR-T3 was correlated with the round-trip accuracy (Fig. 3b). Analysis of test cases showed that a cut-off value of 90% had to be applied to select meaningful validated enzymatic retrosynthetic steps.

**Fig. 4:**
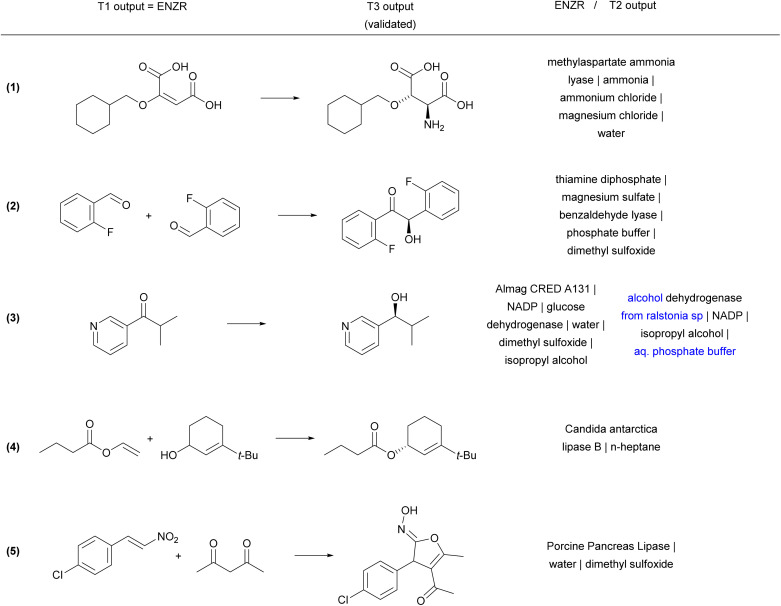
Examples of correctly predicted enzymatic single-step retrosynthesis by ENZR-TTL from the ENZR test set. The confidence scores of T3 are >99.5% in all cases. Enzyme names from the T2 output that differ from the database entry are highlighted in blue.

Reaction examples from the ENZR test set illustrate the performance of ENZR-TTL in terms of single-step retrosynthesis. In many cases, T1 predicts the same SM as recorded in the ENZR dataset, T2 predicts the identical or almost identical enzyme description (with enzyme name, additive and solvents), and T3 predicts the correct P (Fig. 4 and S5†). These include enantioselective reactions with non-biochemical substrates (reaction (1)),^62^ cofactors (reaction (2))^63^ and cofactor regeneration systems (reaction (3),^64^ here with a different T2 output), as well as lipase-catalyzed reactions such as kinetic resolutions by acylation (reaction (4))^65^ and heterocycle formations exploiting the catalytic promiscuity of lipases (reaction (5)).^66^

**Fig. 5:**
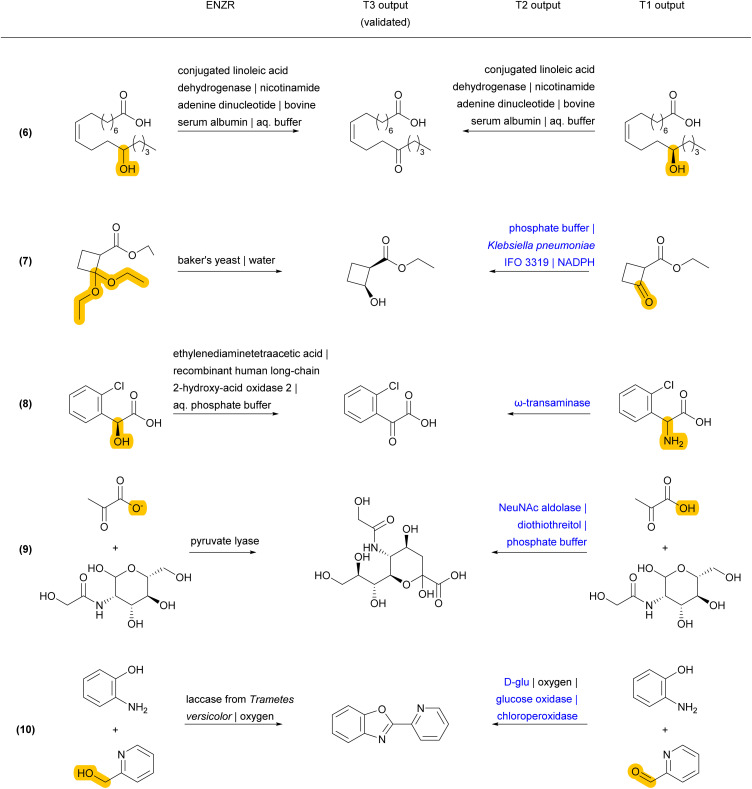
Examples of ENZR-TTL retrosynthetic steps from the ENZR test set validated by T3 involving different precursors and/or enzymes than those in ENZR. Structural differences between SM database entry and T1 output are highlighted in orange and enzyme names from T2 output that differ from the database entry are highlighted in blue.

Validated retrosyntheses by ENZR-TTL include cases where the SM output by T1 and sometimes the enzyme name output by T2 are different from those recorded in ENZR, with interesting cases of reactions involving ketones and aldehydes as SM or P (Fig. 5 and S6†). In one case, the T1 output specifies alcohol chirality for a fatty acid alcohol dehydrogenase reported to be non-enantioselective (although without providing primary data, reaction (6)),^67^ whereby T1 probably infers alcohol chirality from other alcohol dehydrogenases. In another case, a chiral cyclobutanol is proposed by ENZR-TTL to be obtained by reduction of the parent ketone by a microbial dehydrogenase, while the database case involves baker's yeast and a ketal precursor of the cyclobutanone in aqueous pH 2, under which conditions the ketal spontaneously hydrolyzes to give the ketone (reaction (7)).^68^ Furthermore, a (2-chlorophenyl)-ketoacid recorded in ENZR to be formed by enzymatic oxidation of the corresponding mandelic acid,^69^ is predicted by ENZR-TTL to stem from a transaminase reaction from the parent phenylglycine, a known type of biotransformation (reaction (8)).^70^

Some discrepancies between ENZR data and ENZR-TTL output are caused by database entry mistakes and illustrate the self-correcting ability of the transformer model approach. For example, *N*-acetylneuraminic acid is incorrectly recorded in ENZR as involving a “pyruvate lyase” due to an enzyme naming mistake in the corresponding publication (reaction (9)).^71^ For this reaction ENZR-TTL correctly predicts that the enzymatic conversion of SM (*N*-acetyl-mannosamine and pyruvic acid) is carried out by the enzyme NeuNAc aldolase.^72^ Similarly, the oxidative condensation of 2-pyridylmethanol with 2-aminophenol listed in Reaxys as an enzymatic process and recorded in ENZR (reaction (10)) actually involves TEMPO (2,2,6,6-tetramethylpiperidine-1-oxyl) as a chemical oxidant, which is recycled by air oxidation using laccase as enzyme but was not recorded in Reaxys.^73^ Here, ENZR-TTL proposes pincolinaldehyde and 2-aminophenol as SM and a true enzymatic process using glucose oxidase and chloroperoxidase. This bi-enzymatic process has been reported for the related oxidative condensation of benzaldehyde and several *para*-substituted benzaldehydes with 2-aminophenol to form benzoxazoles.^74^

**Fig. 6:**
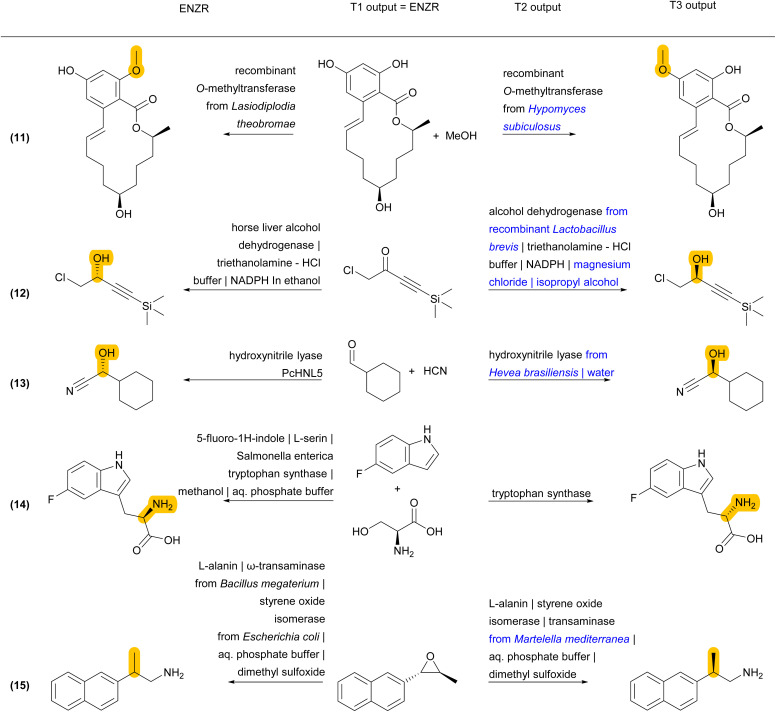
Examples of ENZR-TTL prediction involving a correct SM prediction by T1 but a different enzyme choice by T2 and therefore a different product P compared to the database entry from the ENZR test set. Structural differences between P from database entry and T3 output are highlighted in orange and enzyme names from T2 output that differ from the database entry are highlighted in blue.

Finally, some incorrect cases involve a correct SM prediction by T1, but a different choice of enzyme by T2, resulting in a valid biotransformation but a different product P predicted by T3, and a non-validated reaction in terms of round-trip accuracy of ENZR-TTL (Fig. 6 and S7†). For example, the correct phenolic SM is predicted by T1 for the formation of an *O*-methylated macrolactone (reaction (11)). However, T2 selects a different *O*-methyl transferase enzyme with a different regioselectivity, and therefore T3 predicts a different regioselectivity for the methylation. Note however that the proposed product is the correct one for the selected enzyme, as recorded in the same original publication focusing on tuning *O*-methylation regioselectivity.^75^ In a related case of a chiral propargylic alcohol stemming from reduction of the corresponding ketone by an alcohol dehydrogenase, T1 predicts the correct SM but a change of enzyme choice by T2 results in a T3 prediction of P with the opposite enantioselectivity, which is correct for the selected enzyme but incorrect relative to database entry (reaction (12)).^76^ A similar different enzyme choice by T2 resulting in an enantiomeric P correctly predicted by T3 also occurs for the addition of hydrogen cyanide to cyclohexane carbaldehyde catalyzed by two different hydroxynitrile lyases (reaction (13)).^77,78^

In a related case involving tryptophan synthase, T1 predicts the correct SM, T2 the correct enzyme, and T3 the correct l-enantiomer, however the database entry lists the d-enantiomer, which was obtained by coupling tryptophan synthase with a stereoinversion cascade involving two enzymes that were not listed in the database entry (reaction (14)).^79,80^ In a similar enzymatic cascade yielding 2-(2-naphthyl)propylamine from an epoxide precursor, T1 predicts the correct epoxide SM but combines styrene oxide isomerase with a different transaminase producing the (*R*)-enantiomeric P. By contrast, the database entry for P has an undefined stereochemistry, probably because the parent publications tested various transaminases with different enantioselectivities (reaction (15)).^81,82^

Taken together, the above analysis showed that biocatalytic retrosynthesis predictions by ENZR-TTL were generally relevant and sometimes even corrected inaccuracies in database entries. Encouraged by these data, we moved on to test multi-step chemoenzymatic retrosyntheses with our TTL approach.

### Chemoenzymatic multistep retrosynthesis with TTLAB

Integrating ENZR-TTL alongside the previously reported USPTO-TTL provided the chemo-enzymatic retrosynthesis prediction system, named TTLAB (Fig. 1). To ensure the reliability of the enzymatic steps selected by TTLAB, a confidence score filter of 90% was applied to ENZR-T3. This filter eliminated chemically incorrect enzymatic retrosynthetic steps which would otherwise be selected by the tree-search because they achieved a high RPScore due to a high degree of molecular simplification.

**Fig. 7:**
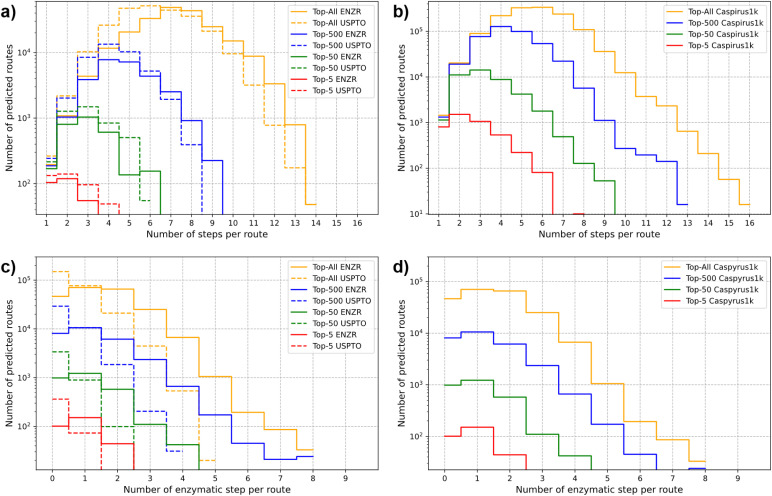
Analysis of synthetic routes predicted by TTLAB on product molecules from the USPTO and ENZR test sets. The route count as function of (a and b) the number of steps per route or (c and d) the number of enzymatic steps per route is given for the different top-N categories.

We challenged TTLAB to propose retrosyntheses for 100 product molecules from the USPTO test set, 80 product molecules from the ENZR test set, and 1000 molecules from the Caspyrus dataset.^83,84^ A retrosynthesis was judged successful whenever the reaction sequence went back to a SM molecule available in the BB set, which consisted of 534 058 commercially available compounds (see Methods for details). TTLAB proposed synthetic routes for 88 of the 100 USPTO test set product molecules, 61 of the 80 ENZR test set product molecules, and 852 of the 1000 molecules of the Caspyrus dataset, and in almost all cases at least one of the proposed routes contained at least an enzymatic step (Table S3†). For TTLAB-predicted syntheses of USPTO and Caspyrus molecules, approximately 8% and 9% of the proposed steps were enzymatic. This percentage ranged from 17% to 50% for TTLAB predicted syntheses of ENZR molecules considering either all proposed syntheses or only top-scoring ones (Table S4†). The ability of TTLAB to identify short chemo-enzymatic synthetic routes was well visible when analyzing the number of steps per route as well as the number of enzymatic steps per route among the top-5, top-50, top-500 or all routes for USPTO, ENZR and Caspyrus molecules (Fig. 7).

**Fig. 8:**
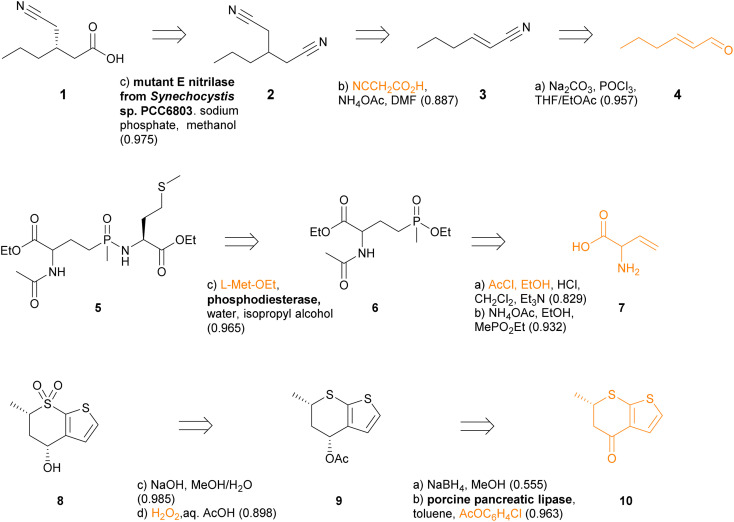
Top RPScoring retrosyntheses predicted by TTLAB including at least one enzymatic step for three ENZR test set products. The confidence score of each predicted step is indicated in parentheses. Starting materials in the commercial BB set are written in orange.

The chemoenzymatic routes predicted by TTLAB are well illustrated by three examples from the ENZR test set, for which we show in each case the best RPScoring route including at least one enzymatic step (Fig. 8). The first example is the predicted synthesis of the chiral cyanocarboxylic acid 1, which was reported as the product of the enantioselective mono-hydrolysis of the prochiral dinitrile 2 by a mutant nitrilase enzyme.^85^ TTLAB predicts the identical biotransformation as the first retrosynthetic operation, and proposes to assemble dinitrile 2 by Michael addition of cyanoacetic acid to unsaturated nitrile 3 and decarboxylation. Finally, TTLAB proposes to prepare nitrile 3 from the parent aldehyde 4, which is a well-known type of transformation however using different reagents.^86^

The second example is the predicted synthesis of the phospha-C-peptide 5, which was reported to be formed by coupling l-methionine ethyl ester with ethyl phosphinate 6 catalyzed by a phosphordiesterase.^87^ TTLAB proposes the identical last step using the same enzyme. Since phosphinate 6 is not present in the commercial BB set, TTLAB further proposes a synthesis from vinyl glycine 7 by *N*-acetylation and esterification, done as a single step, followed by addition of ethyl methylphosphinate to the double bond. The latter reaction had been reported to prepare l-phosphinothricin, a naturally occurring herbicidal amino acid, however TTLAB omits to list the required radical initiator *tert*-butyl *per*-2-ethylhexanoate.^88^

The third example is chiral sulfone 8, which TTLAB would prepare by deacetylation and sulfide oxidation of intermediate 9 using known chemistry.^89^ Intermediate 9 would be formed by diastereoselective enzymatic acetylation of the parent alcohol by porcine pancreatic lipase using *p*-chlorophenylacetate as acylating agent, a biotransformation reaction known from the test set.^90^ This parent alcohol would be formed by non-stereoselective reduction of ketone 10 using sodium borohydride. This reduction is predicted with low confidence by TTLAB because this reaction can in fact be performed stereoselectively using LiAlH_4_.^91^ Indeed, when the condition of an enzymatic step is not imposed, TTLAB readily proposes, as the second best RPScoring route, a two-step chemical synthesis of 8 from 10 by stereoselective reduction followed by thioether oxidation to the sulfone.

**Fig. 9:**
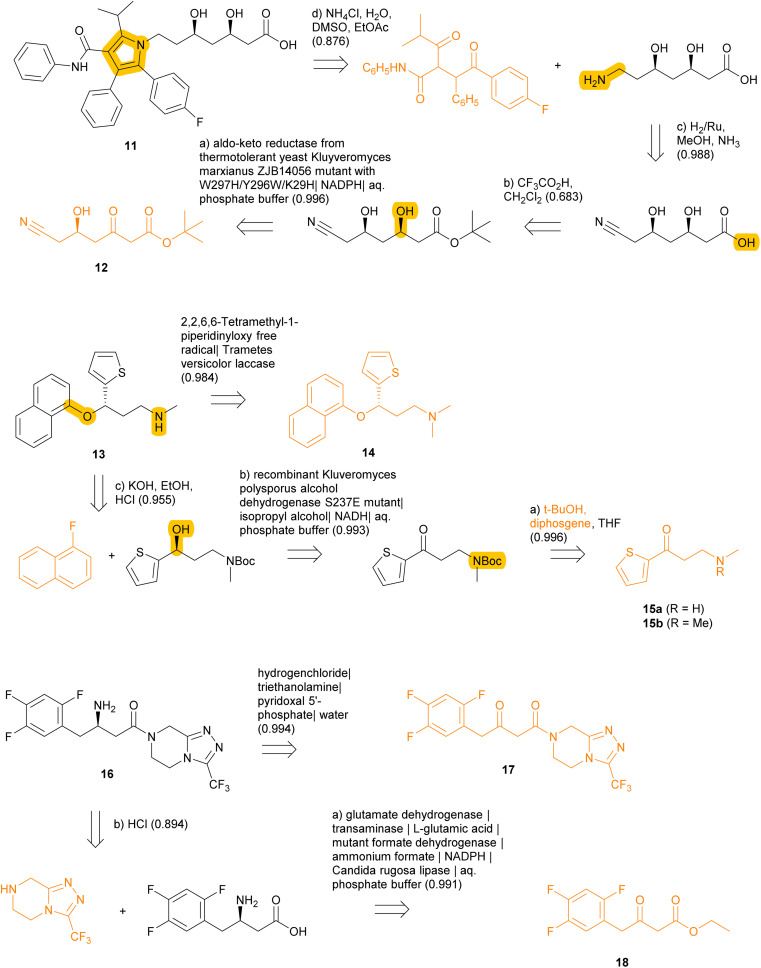
Retrosyntheses of atorvastatin (11), (*S*)-duloxetine (13) and sitagliptin (16) proposed by TTLAB. Reactive bonds and starting materials in the commercial BB set are drawn in orange. The confidence scores of individual retrosynthetic steps are indicated in parentheses after the predicted reagents.

We further exemplify TTLAB in the prediction of chemoenzymatic retrosyntheses for three drugs with known chemoenzymatic routes (Fig. 9). In these cases, TTLAB often identifies steps that are part of the training sets. For the first case of the cholesterol-lowering drug atorvastatin 11, our algorithm proposes as best RPScoring route the acidic deprotection of the corresponding *tert*-butyl ester, which is a commercial building block. Imposing at least one enzymatic step results in a four-step sequence from a linear chiral keto-ester precursor 12, for which the first step is an enzymatic reduction by an aldo-keto reductase which was evolved precisely for this purpose and is present in the TTLAB training set.^92^ The overall TTLAB route design is similar to the chemoenzymatic process developed for this drug involving an enzymatic enantioselective reduction of ethyl cyanoacetoacetate as initial step.^93^

In the second case of the antidepressant (*S*)-duloxetine 13, the top-RPScoring route with at least one enzymatic step predicted by TTLAB is the single-step demethylation of the commercial *N*,*N*-dimethyl analog 14 catalyzed by a laccase, and the second best is a three-step sequence involving Boc protection of the achiral ketone precursor 15a, followed by enantioselective reduction with an alcohol dehydrogenase and arylation of the resulting alcohol with fluoronaphthalene. This route is similar to the published chemoenzymatic synthesis of this drug starting with *N*,*N*-dimethylketone 15b,^94^ also proposed by the ASKCOS chemical CASP tool with the help of manual intervention to introduce biocatalytic steps.^95^

In the third case of the DDP4 inhibitor sitagliptin (16) used to treat type II diabetes, TTLAB identifies a single-step enzymatic enantioselective retrosynthesis from the commercial β-ketoamide 17 using a transaminase. Although TTLAB only names the PLP cofactor in the reagents, this step is present in the ENZR training set using a transaminase that has been engineered for the synthesis of this drug.^96^ The second best RPScoring route is a similar two step sequence from the commercial ketoester 18 involving an enzymatic enantioselective reductive amination followed by amide bond formation. Note that the enzymatic step is part of the ENZR training set and uses the exact same combination of four enzymes for this biotransformation,^97^ illustrating that transformer model ENZR-T2 memorizes enzyme textual description with high accuracy.

The above analysis and application examples show that TTLAB can propose short chemoenzymatic retrosyntheses for various target molecules. It should be noted that enzymatic steps are selected by TTLAB only when the reaction is closely related to a training set reaction, reflecting the fact that biocatalytic reactions are often highly specific for certain types of starting materials and are intrinsically poorly generalizable.

### Comparison with other chemo-enzymatic CASP tools

To compare TTLAB with other chemo-enzymatic retrosynthesis tools, we subjected the six target molecules discussed above (1, 5, 8, 11, 13 and 16, Fig. 8 and 9) to the IBM RXN for Chemistry retrosynthesis prediction tool in “Automatic mode” using the “enzymatic mode 2022-05-31” model and “high quality” tuning, which uses the reported transformer model.^36^ We also tested the template-based tool ASKCOS as available online using either the “reaxys_biocatalysis” and “reaxys” models combined, or just the “reaxys_biocatalysis” model alone,^45^ as well as the recently reported chemo-enzymatic version of BioNavi with the “Default settings” preset, allowing both “Bio-building blocks” and “Chemo-building blocks”, and combining “Enzymatic synthesis” and “Non-enzymatic synthesis”.^35,98^

The IBM RXN for chemistry provided retrosyntheses for all six target molecules, however none of the retrosyntheses contained any enzymatic steps in the sequences and the sequences went back to chiral building blocks as source of chirality (Table 2 and Fig. S8–S13†). The difficulty of this tool in identifying biocatalytic steps might reflect the fact that it uses biochemical reaction data and very different molecule types as discussed above (Fig. 2). On the other hand, ASKCOS only predicted retrosyntheses successfully for 13 and 16. In these two cases, at least one route contained enzymatic steps and the routes were similar to those coming from TTLAB, with enzymatic steps involved in establishing stereochemistry in two cases (Table 2 and Fig. S14–S21†). This similarity might reflect the fact that ASKCOS also exploits literature data on biotransformations collected from Reaxys, although in a different manner that TTLAB. BioNavi only produced retrosyntheses for 1 and 5, however these were short and included biocatalytic steps (Fig. S22–S24†).

**Table 2:** Summary of the number of chemical steps (C) and number of biocatalysis steps (B) for each target molecule using various combined chemical and biocatalysis tools

Target molecule^a^	TTLAB	IBM RXN^36^	ASKCOS^45^	BioNavi^98^
1	2C + 1B	8C	—	3C + 1B, 3C^b^
5	1C + 1B	7C	—	2C, 1B
8	1C + 1B	1C	—	—
11	3C, 1B	1C	—	—
13	1B, 2C + 1B	2C	1C, 2C + 1B	—
16	1B, 1C + 1B	4C	1C, 1B, 2B	—

a See Fig. 8 and 9 for TTLAB retrosyntheses, Fig. S8–S13 for IBM RXN for chemistry retrosyntheses, Fig. S14–S21 for ASKCOS retrosyntheses, and Fig. S22–S24 for BioNavi retrosyntheses.

b another 6 routes were proposed by BioNavi for 1 with up to 5 steps, however without enzymatic steps.

Although the three chemoenzymatic CASP tools tested did not perform as well than TTLAB in the examples discussed above, one cannot generalize and each retrosynthesis should be analyzed in detail for feasibility. In that respect, it must be noted that for chemical steps IBM RXN for chemistry ouputs the reagents as part of the starting materials and describes the reaction class for each transformation, thereby providing an information comparable to the output of TTLAB. For enzymatic steps however for which TTLAB provides enzyme names, IBM RXN for chemistry only provides EC numbers, which can be insufficient to choose a particular enzyme in cases such as lipases and alcohol dehydrogenases for which substrate tolerance and stereoselectivity are highly variable. On the other hand, ASKCOS does not provide reagents or enzyme names but simply links to Reaxys references, which have to be searched manually to identify the proper reaction conditions. Finally, BioNavi informs whether a given steps is enzymatic or non-enzymatic and upon request connects to a list of reagents or enzymes, which again is an output similar to TTLAB. Note that each tool uses a slightly different set of commercial building blocks, which may influence the ability to propose retrosynthetic routes as well as route length depending on the availability of advanced intermediates in the building block set.

## Conclusion

In summary, our work integrates biocatalysis in a computer-assisted synthesis planning (CASP) system, going towards greener and more sustainable chemistry. We achieved this by introducing a dual multistep retrosynthesis prediction system, integrating both chemical and biocatalytic steps in the form of two triple transformer loops, namely our previously reported TTL trained on USPTO reactions for chemical steps (USPTO-TTL),^53^ and a related enzymatic ENZR-TTL trained on an updated version of our ENZR dataset of biotransformations extracted from Reaxys.^52^ ENZR-TTL makes use of a new model to mark potential biocatalytic disconnection sites (ENZR-Autotag) in product molecules and consists of three new transformers to predict and validate possible retrosynthetic biotranformations. The competitive framework, driven by the route penalty score (RPScore), drives the selection of optimal steps by our best-first tree search, incorporating both catalytic steps to generate mixed synthesis routes. In the successful routes selected by TTLAB, 8–17% of the steps (depending on the molecular dataset) are enzyme-catalyzed reactions, suggesting that our tool can valuably contribute to green process design. Our results not only showcase the tool's capabilities in proposing viable solutions for drug-like molecules but also establish it as a valuable resource for synthesis design. The continuous enrichment of data in Reaxys promises ongoing enhancements in enzymatic capabilities, progressively going towards enzymatic synthesis.

## Data availability

Code and instructions to compute multistep retrosynthesis as well as the code to tag reactive sites are available on our GitHub repository: https://github.com/reymond-group/MultiStepRetrosynthesisTTL. The original USPTO dataset can be found at https://doi.org/10.6084/m9.figshare.5104873.v1. The derived version of the USPTO dataset of Thakkar *et al.* can be found in their preprint.^54^ The Reaxys enzymatic dataset is a licensed commercial database that cannot be made available.

## Author contributions

DK designed and carried out the study and wrote the paper, JLR designed and supervised the study and wrote the paper.

## Conflicts of interest

The authors declare that they have no competing interests.

## Supplementary Material

SC-OLF-D4SC02408G-s001
