# Supplementary material for: Chemoenzymatic multistep retrosynthesis with transformer loops
Source: Chem Sci. 2024 Oct 8;15(43):18031–47. doi: 10.1039/d4sc02408g (PMC11474389; doi:10.1039/d4sc02408g)
Supplement: SC-015-D4SC02408G-s001 [file SC-015-D4SC02408G-s001.pdf]

Supplementary Information (SI) for Chemical Science.
This journal is © The Royal Society of Chemistry 2024

Supporting Information for

Chemoenzymatic Multistep Retrosynthesis with Transformer Loops

David Kreutter[a)] and Jean-Louis Reymond*[a)]

[a)] *Department of Chemistry, Biochemistry and Pharmaceutical Sciences, University of Bern, Freiestrasse 3, 3012 Bern, Switzerland*

e-mails: david.kreutter@unibe.ch
jean-louis.reymond@unibe.ch

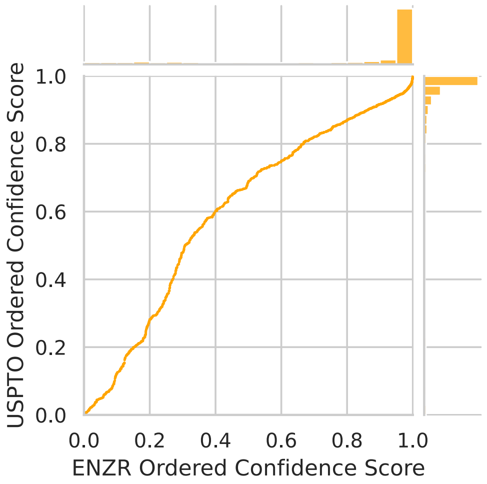

Figure S1. Ordered confidence scores of the ENZR-TTL T3 as function of the ordered confidence scores of USPTO-TTL T3 on the ENZR test set and USPTO test set respectively.

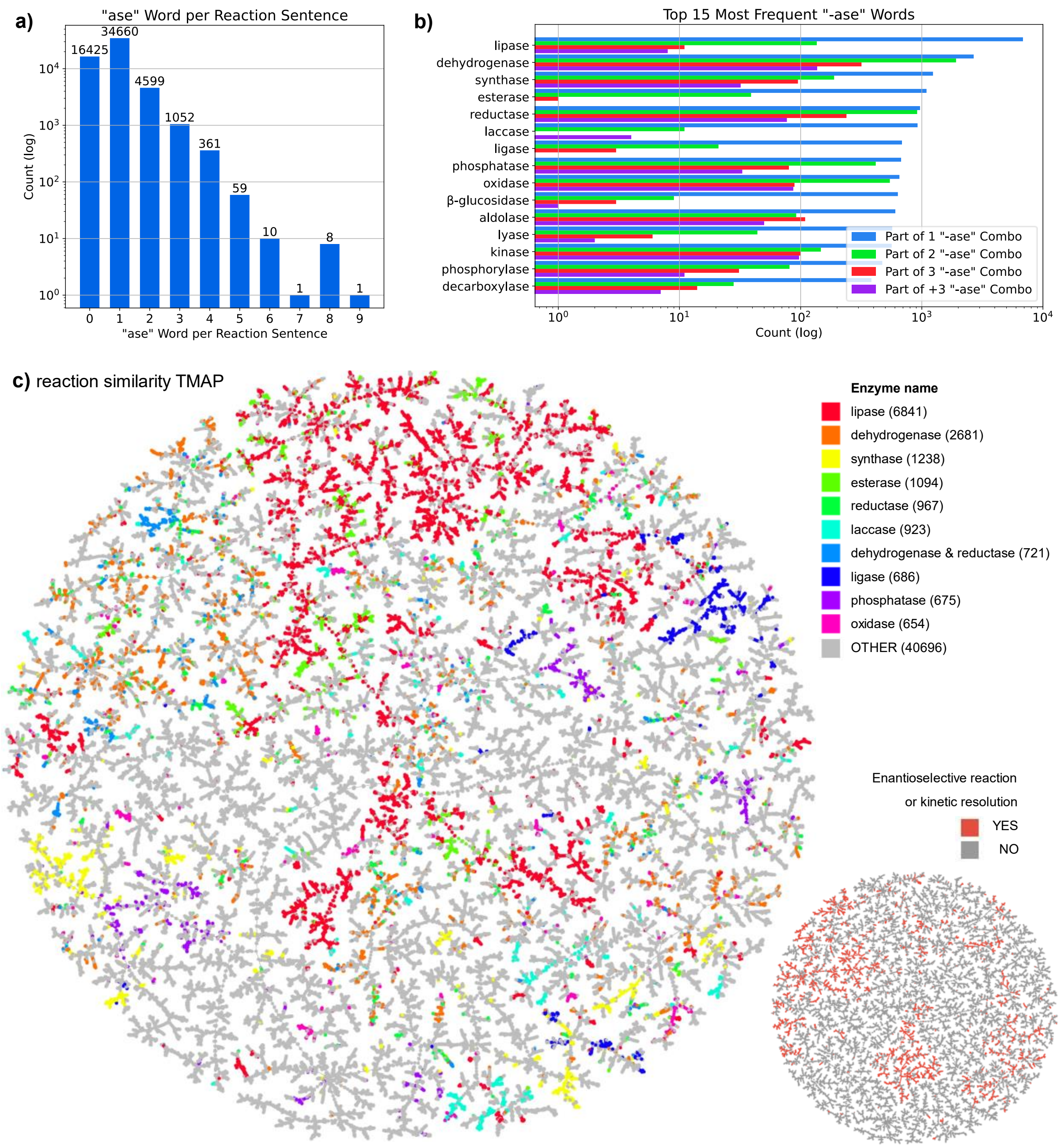

Figure S2. Analysis of the ENZR dataset. (**a**) Number of reactions depending on how many "-ase" words are present in the sentence for a given reaction. (**b**) Frequency of the top 15 "-ase" words depending on the count of enzyme name per reaction. (**c**) TMAP of reactions similarity color-coded by the 10 most frequent "-ase" words as listed in Fig. 2b. combinations. The "other" category groups reactions with "-ase" words other than the top 10 "-ase" words or reaction containing infrequent "-ase" word combinations. Insert lower right: TMAP highlighting enantioselective and kinetic resolution reactions.

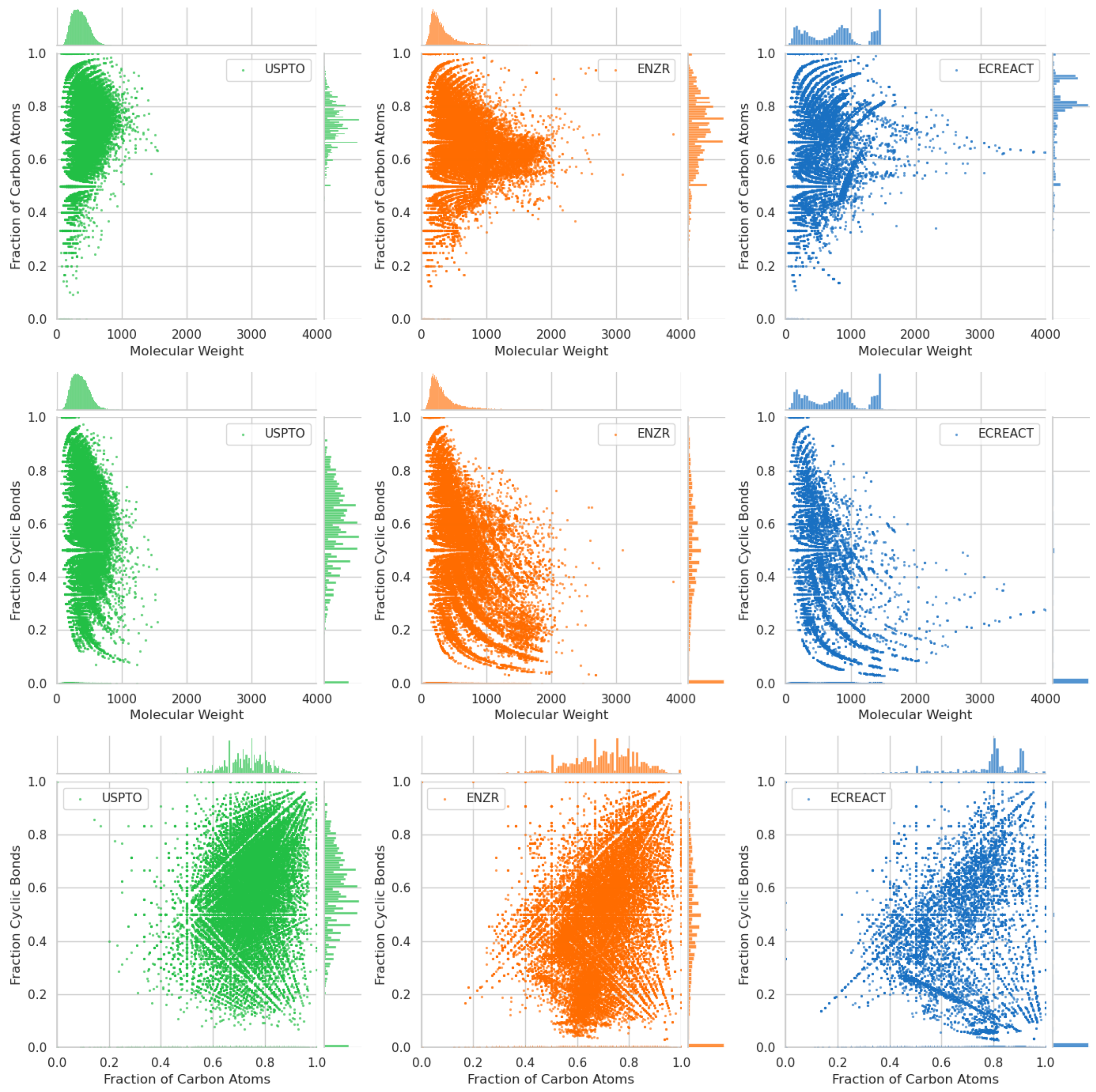

Figure S3. Analysis of the USPTO (green), ENZR (orange) and ECREACT (blue) datasets in forms of scatter plots. First line: Fraction of C-atoms vs. MW. Second line: Fraction cyclic bonds vs. MW. Third line: Fraction cyclic bonds vs. Fraction of C-atoms.

Table S1. Details of top-1 round-trip accuracy by ENZR-TTL single step retrosyntheses on the 2858 molecules of the ENZR test set.

	Round-trip validated by T3	Not validated by T3
Ground-truth predicted SM	49.41%	23.13%
Not ground truth predicted SM	9.55%	17.91%

Table S2. Details of top-1 round-trip accuracy by USPTO-TTL single step retrosyntheses on a sample of 3000 molecules from the USPTO test set.

	Round-trip validated by T3	Not validated by T3
Ground-truth predicted SM	60.57%	6.97%
Not ground truth predicted SM	20.73%	11.73%

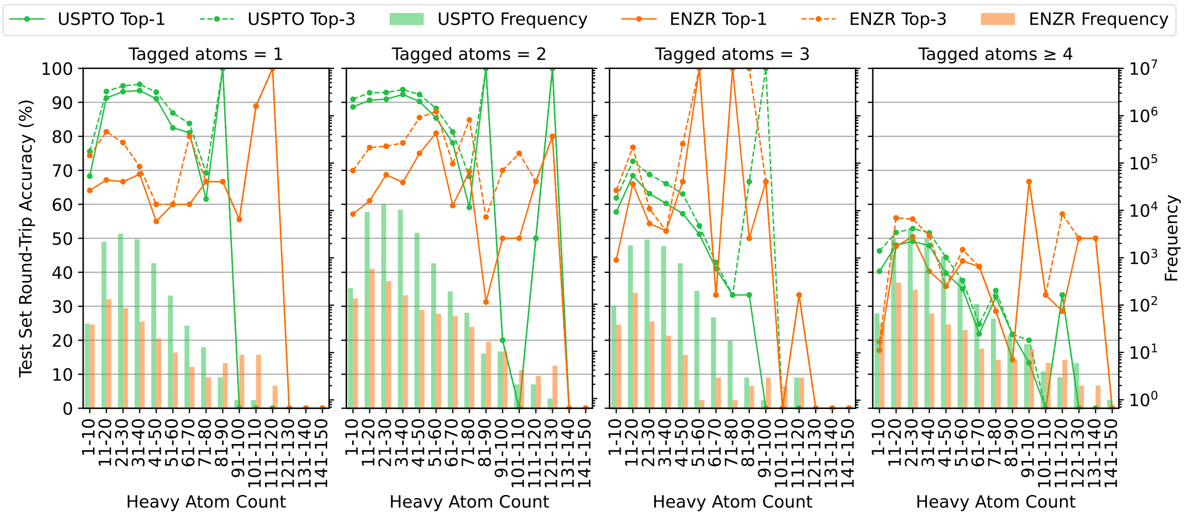

Figure S4. Round-trip accuracies of ENZR-TTL and USPTO-TTL as function of the heavy atom count, for different numbers of tagged atoms, on the target molecules from the ENZR and USPTO test sets respectively. The top-N represents the round-trip accuracy considering multiple examples of enzyme textual descriptions predicted by ENZR-T2 or reagents predicted by USPTO-T2. The bar plots show the frequencies as function of the heavy atom count for both test sets (log-scale right axis).

T1 output = ENZR | T3 output (validated) | ENZR / T2 output

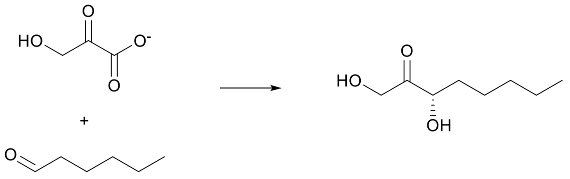

magnesium(II) chloride hexahydrate | thiamine pyrophosphate | transketolase from *Geobacillus stearothermophilus* variant L382F | water

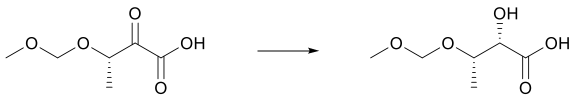

1,4-dihydronicotinamide adenine dinucleotide | *Bacillus stearothermophilus* L-lactate dehydrogenase | *Candida boidinii* Boerhinger formate dehydrogenase | water

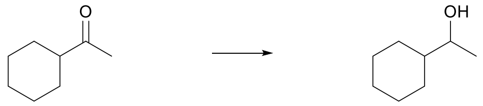

ENZR: 2,3,4,5,6-pentahydroxy-hexanal | *Bacillus megaterium* glucose dehydrogenase | recombinant *Hansenula polymorpha* ketoreductase | nicotinamide adenine dinucleotide | aq. phosphate buffer

T2 output: imine reductase from *Streptomycesipomoeae* | NADPH | methanol

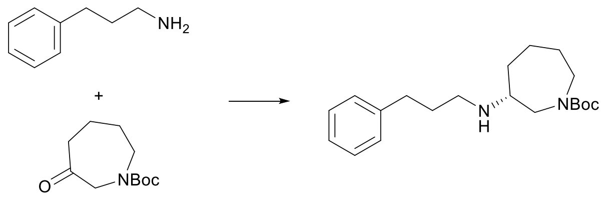

glucose dehydrogenase | beta-nicotinamide adenine dinucleotide phosphate disodium salt | D-glucose|imine reductase from *Actinoalloteichushymeniacidonis* N101T/S240F/F187I/M218A/M244H/A247H/L177I/N251K/H169A/G125K mutant | aq. phosphate buffer | dimethyl sulfoxide

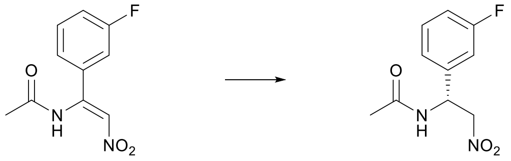

D-glucose | glucose dehydrogenase from *Bacillus megaterium* DSM509 strain | old yellow enzyme 3 from *S. cerevisiae* BY4741 strain | NADP | aq. phosphate buffer | dimethyl sulfoxide

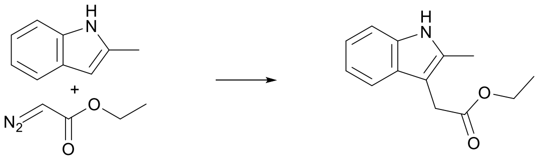

ENZR: *E. coli* cells expressing whale myoglobin(L29F,H64V) | aq. phosphate buffer

T2 output: *E. coli* harboring cytochrome P450-BM3 mutated variant P411BM-CIS V87T H92F L181G N201S L215Q I263M V281L T438C K472T N573D F646S Q674* | aq. phosphate buffer | ethanol | dimethyl sulfoxide

Figure S5. Additional examples of correctly predicted enzymatic single step retrosynthesis by ENZR-TTL. The confidence scores of T3 are >99.5% in all cases. Enzyme names from the T2 output that differ from the database entry are highlighted in blue.

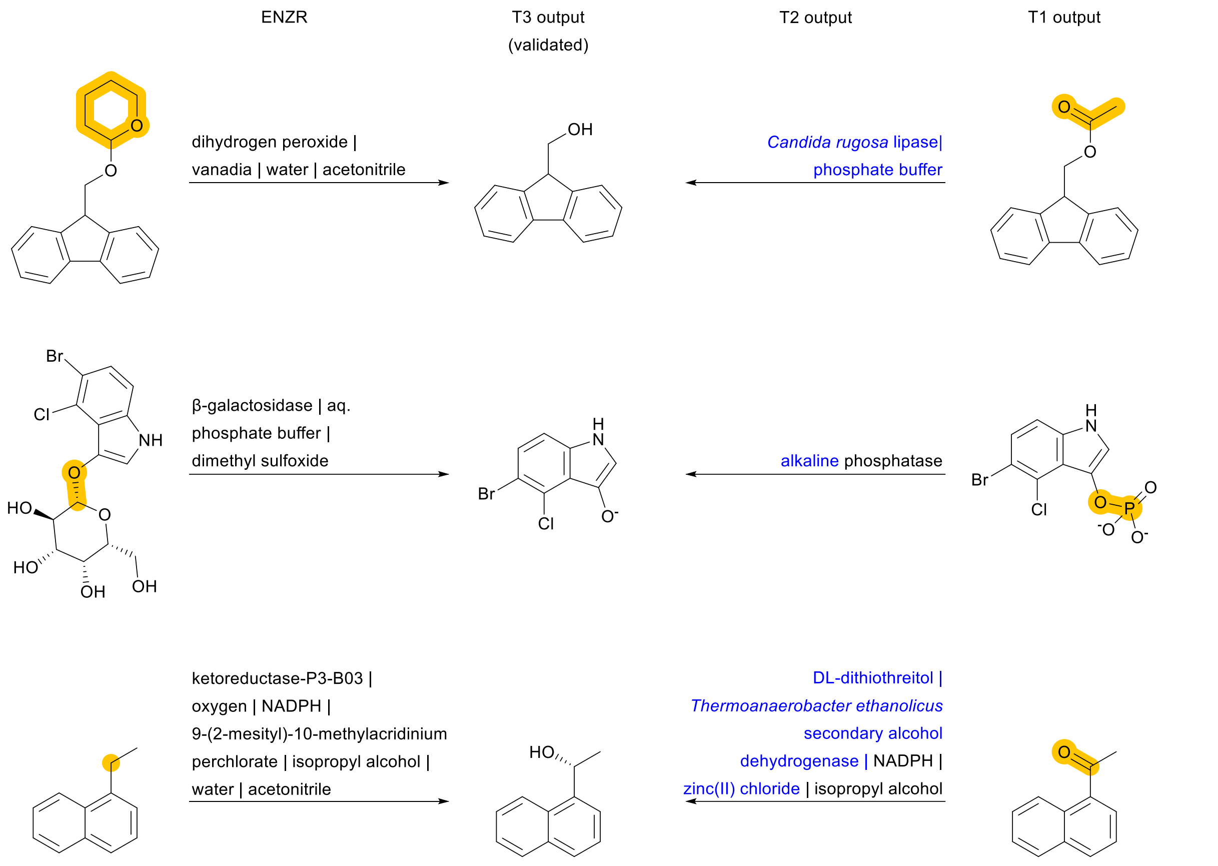

Figure S6. Additional examples of ENZR-TTL retrosynthetic steps validated by T3 involving different precursors and/or enzymes than those in ENZR. Structural differences between SM database entry and T1 output are highlighted in orange and enzyme names from T2 output that differ from the database entry are highlighted in blue.

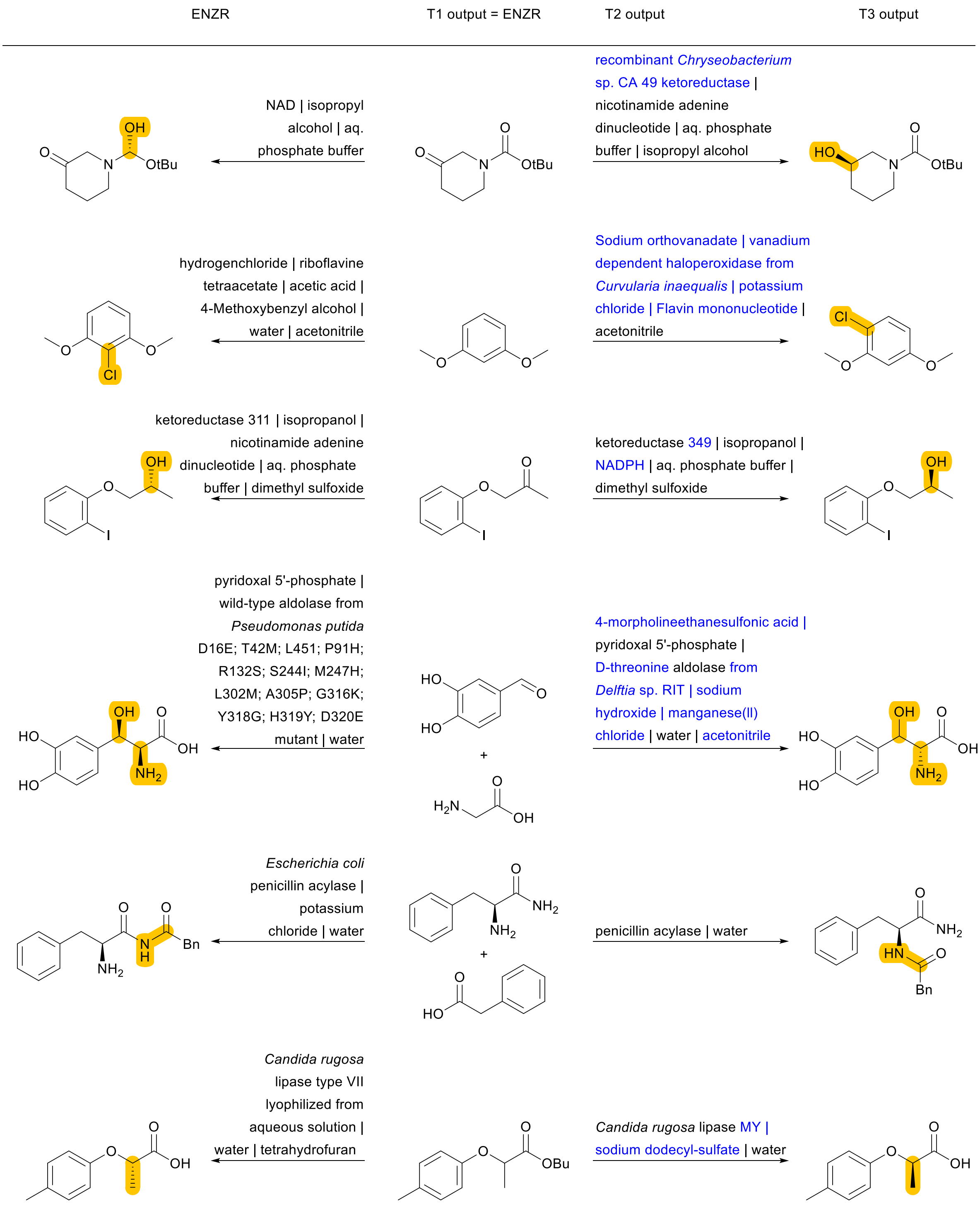

Figure S7. Additional examples of ENZR-TTL prediction involving a correct SM prediction by T1 but a different enzyme choice by T2 and therefore a different product P compared to the database entry.

Table S3. Number (percentage) of product molecule from the test set with solved routes for the selection of 80 molecules from the ENZR test set, for 100 molecules from the USPTO test set, and on the full 1k Caspyrus dataset.

	USPTO test set (100 molecules)	ENZR test set (80 molecules)	Caspyrus-1k (1000 molecules)
Molecules with Route solved	88 (88%)	61 (76%)	852 (85.2%)
Molecules with Route solved with at least one route including an enzymatic step	86 (86%)	60 (75.0%)	782 (78.2%)

Table S4. Fraction of enzymatic reaction steps present in the predicted and solved multistep routes among the top-X route unique steps, ranked according to the RPScore. Tested on 100 USPTO test set, 80 ENZR test set molecules and the 1k Caspyrus dataset.

	for USPTO test set molecules (%)	for ENZR test set molecules (%)	For Caspyrus-1k molecules (%)
Overall	7.88	16.86	8.62
Top-100 RPScoring routes	9.33	21.67	9.77
Top-50 RPScoring routes	9.33	24.79	10.09
Top-10 RPScoring routes	8.65	33.76	10.01
Top-5 RPScoring routes	8.23	38.22	9.97
Top-1 RPScoring routes	7.10	50.00	10.19

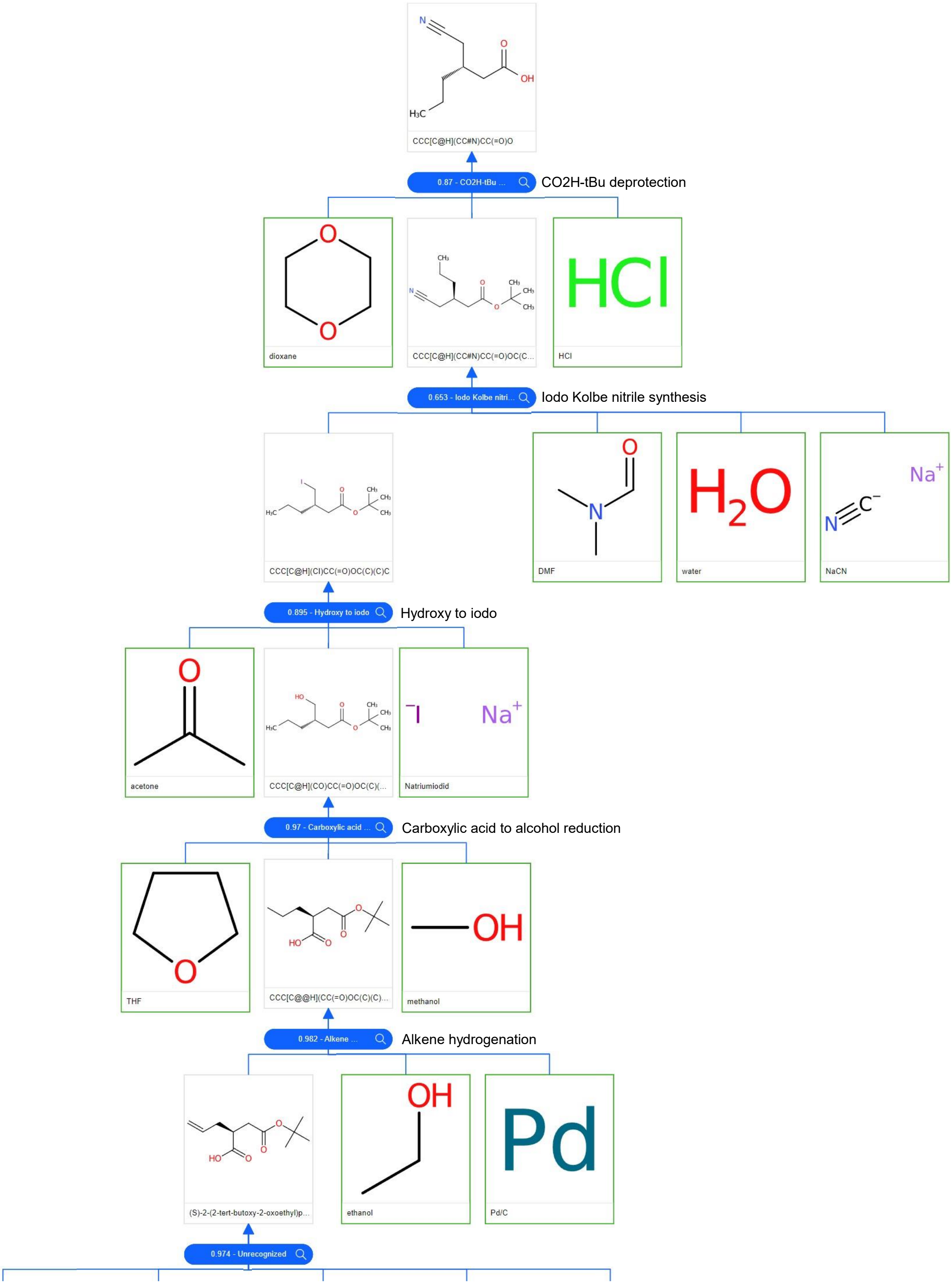

CCC[C@H](CC#N)CC(=O)O
0.87 - CO2H-tBu ...
CO2H-tBu deprotection
dioxane
CCC[C@H](CC#N)CC(=O)OC(C...
HCl
0.653 - Iodo Kolbe nitri...
Iodo Kolbe nitrile synthesis
CCC[C@H](CI)CC(=O)OC(C)(C)C
DMF
water
NaCN
0.895 - Hydroxy to iodo
Hydroxy to iodo
acetone
CCC[C@H](CO)CC(=O)OC(C)(...
Natriumiodid
0.97 - Carboxylic acid ...
Carboxylic acid to alcohol reduction
THF
CCC[C@@H](CC(=O)OC(C)(C)...
methanol
0.982 - Alkene ...
Alkene hydrogenation
(S)-2-(2-tert-butoxy-2-oxoethyl)p...
ethanol
Pd/C
0.974 - Unrecognized

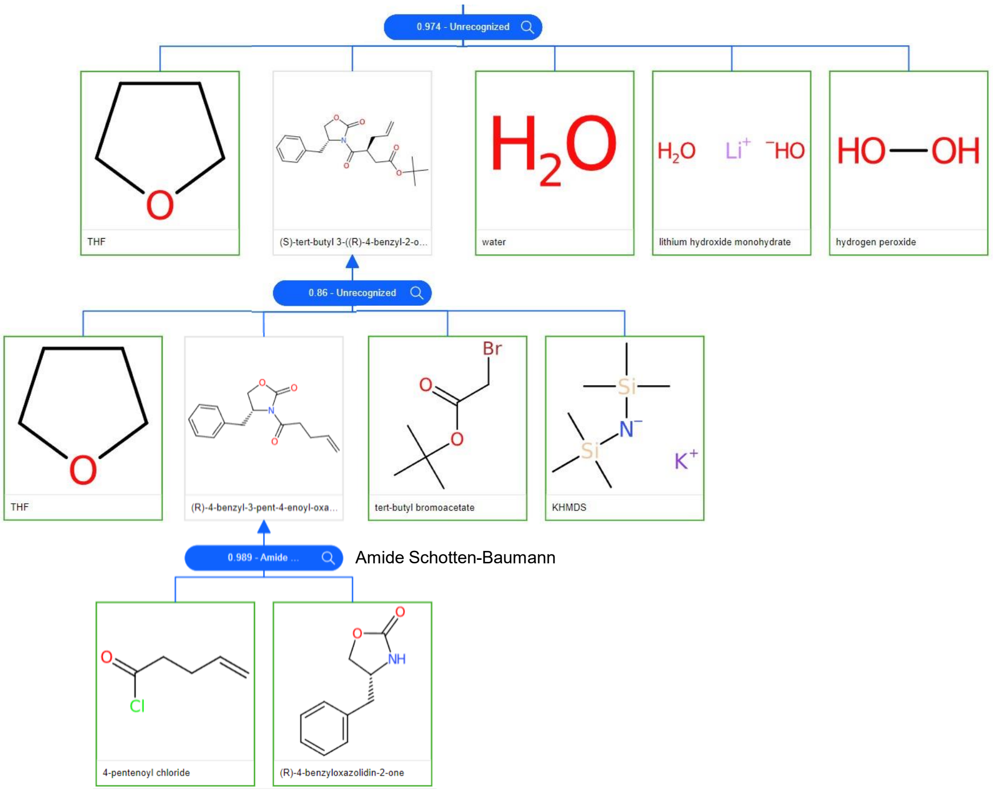

Figure S8. Molecule **1** best scoring route predicted by the IBM RXN for Chemistry retrosynthesis prediction tool in “Automatic mode” using the “enzymatic mode 2022-05-31” model and “high quality” tuning, other settings were left as default.

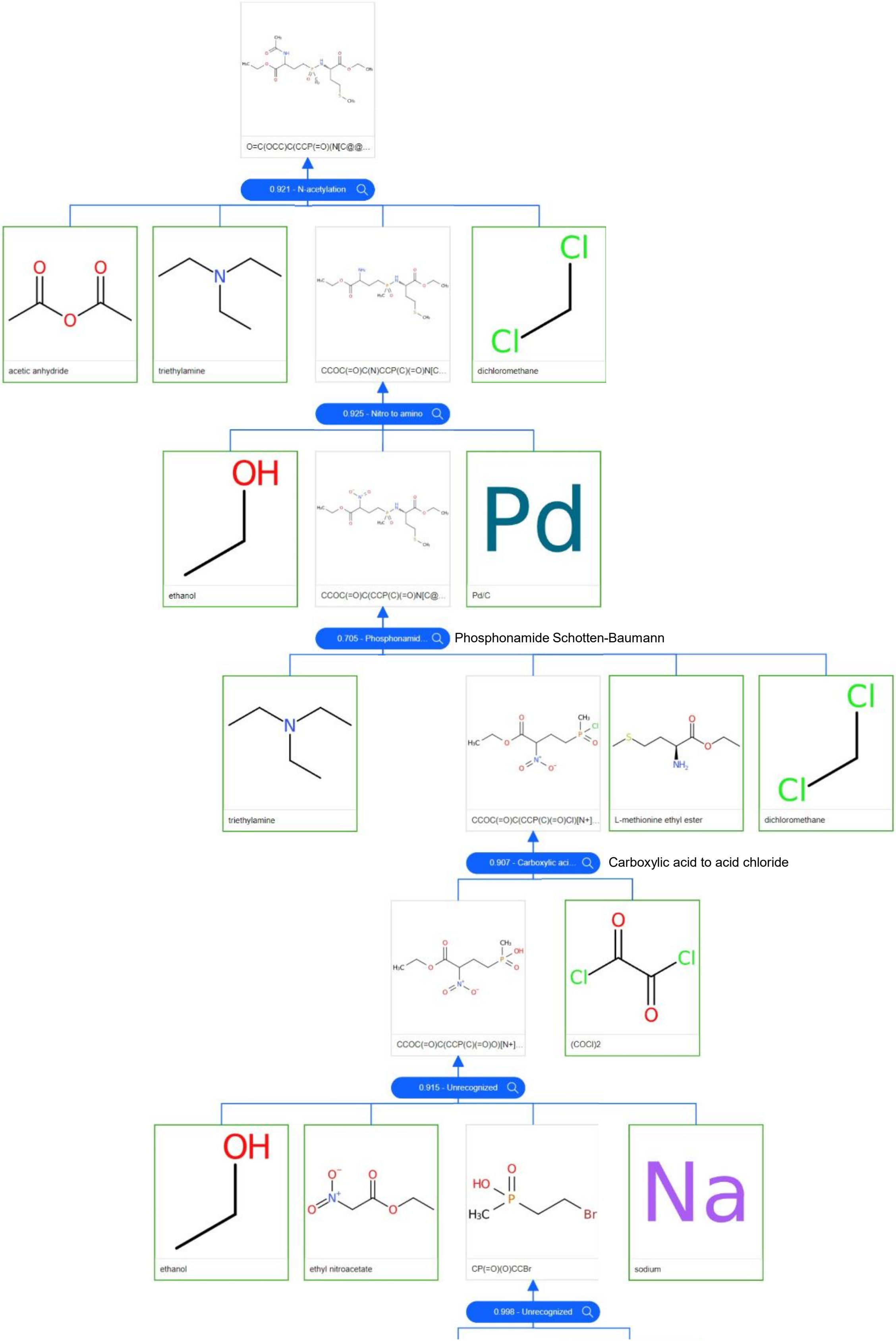
O=C(OCC)C(CCP(=O)(N[C@@...
0.921 - N-acetylation
acetic anhydride
triethylamine
CCOC(=O)C(N)CCP(C)(=O)N[C...
dichloromethane
0.925 - Nitro to amino
ethanol
CCOC(=O)C(CCP(C)(=O)N[C@...
Pd/C
0.705 - Phosphonamid...
Phosphonamide Schotten-Baumann
triethylamine
CCOC(=O)C(CCP(C)(=O)Cl)[N+]...
L-methionine ethyl ester
dichloromethane
0.907 - Carboxylic aci...
Carboxylic acid to acid chloride
CCOC(=O)C(CCP(C)(=O)O)[N+]...
(COCl)2
0.915 - Unrecognized
ethanol
ethyl nitroacetate
CP(=O)(O)CCBr
sodium
0.998 - Unrecognized

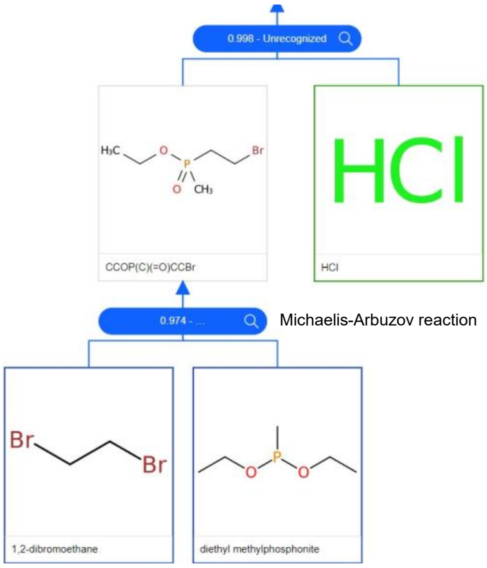

Figure S9. Molecule **5** best scoring route predicted by the IBM RXN for Chemistry retrosynthesis prediction tool in “Automatic mode” using the “enzymatic mode 2022-05-31” model and “high quality” tuning, other settings were left as default.

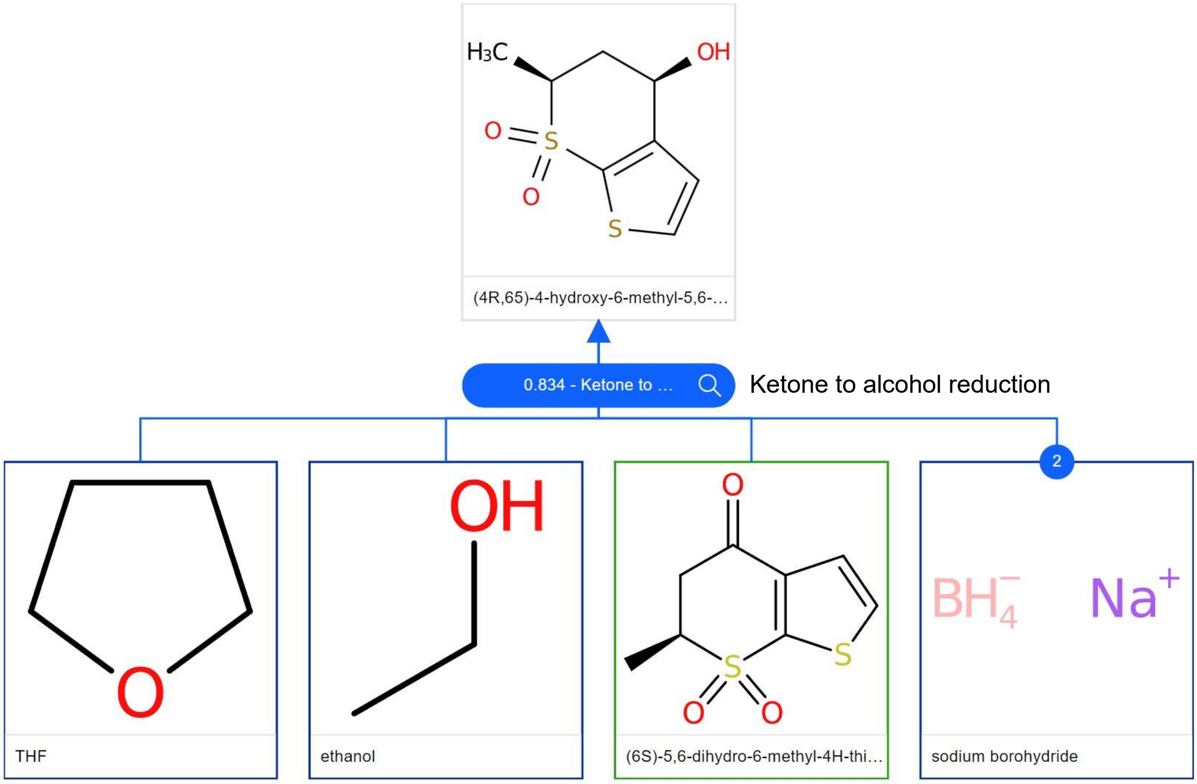

Figure S10. Molecule **8** best scoring route predicted by the IBM RXN for Chemistry retrosynthesis prediction tool in “Automatic mode” using the “enzymatic mode 2022-05-31” model and “high quality” tuning, other settings were left as default.

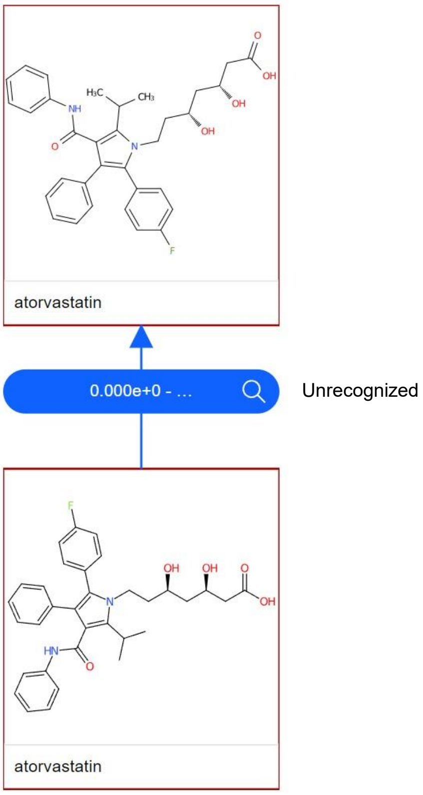

Figure S11. Molecule **11** best scoring route predicted by the IBM RXN for Chemistry retrosynthesis prediction tool in “Automatic mode” using the “enzymatic mode 2022-05-31” model and “high quality” tuning, other settings were left as default.

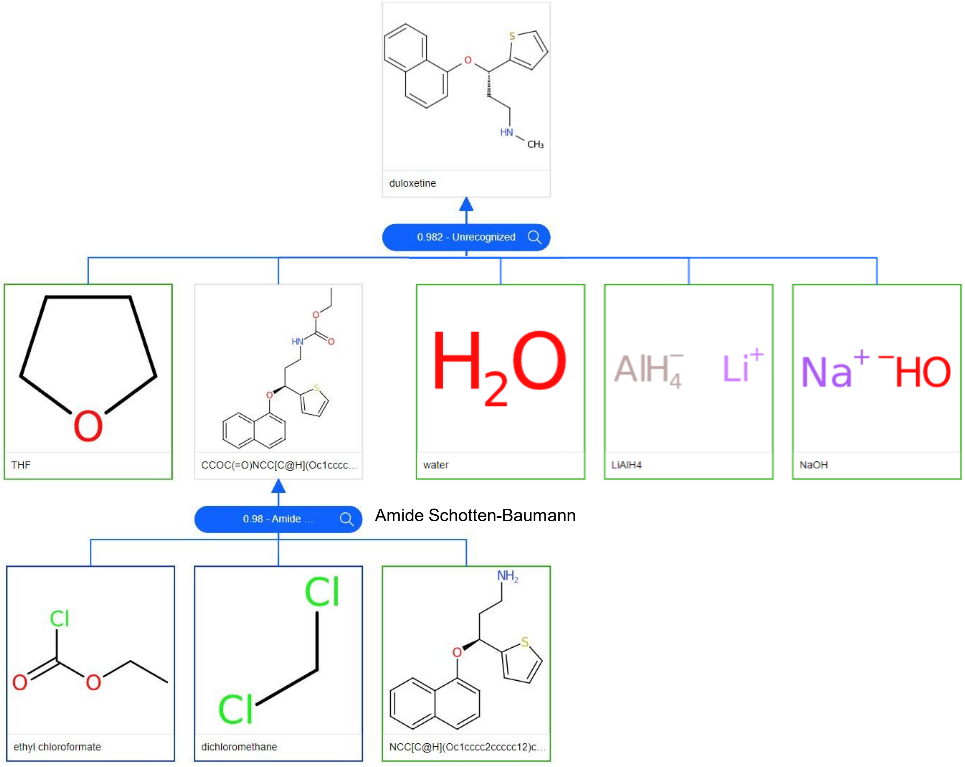

Figure S12. Molecule **13** predicted best scoring route predicted by the IBM RXN for Chemistry retrosynthesis prediction tool in “Automatic mode” using the “enzymatic mode 2022-05-31” model and “high quality” tuning, other settings were left as default.

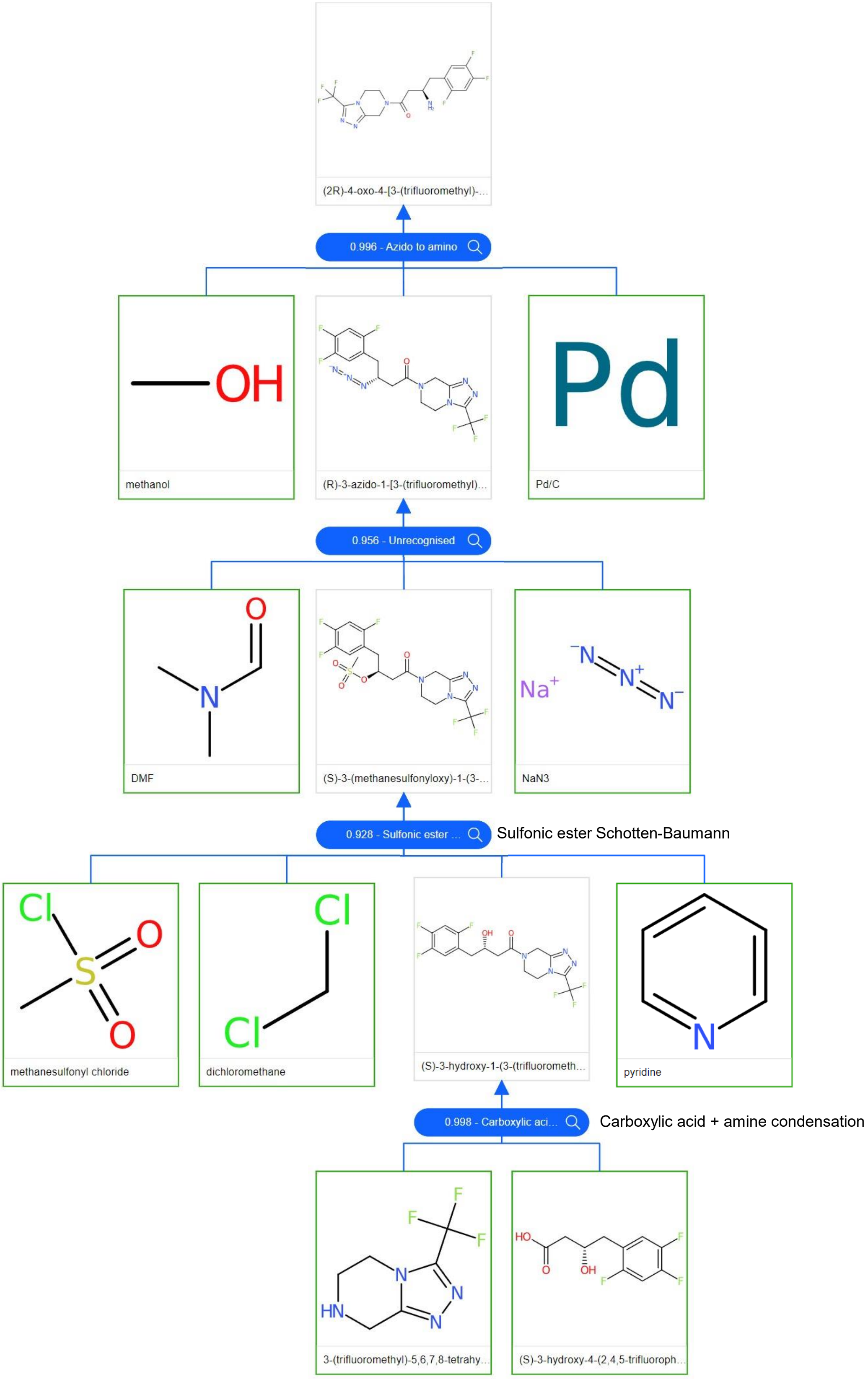

Figure S13. Molecule **16** predicted best scoring route predicted by the IBM RXN for Chemistry retrosynthesis prediction tool in "Automatic mode" using the "enzymatic mode 2022-05-31" model and "high quality" tuning, other settings were left as default.

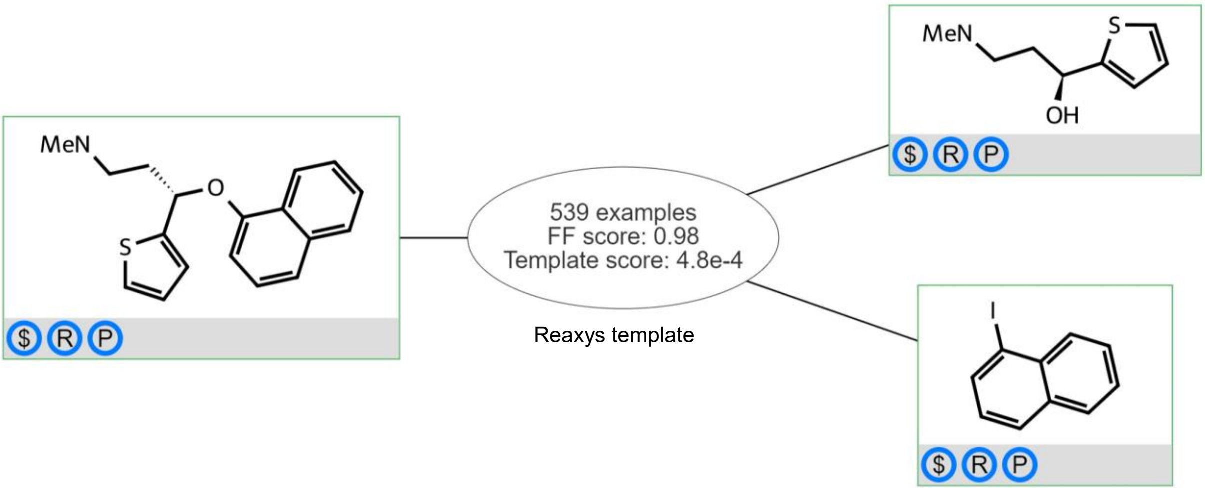

Figure S14. Top 1 scoring route of molecule **13** predicted by the ASKCOS retrosynthesis prediction tool combining the "reaxys_biocatalysis" and the "reaxys" models, other settings were left as default.

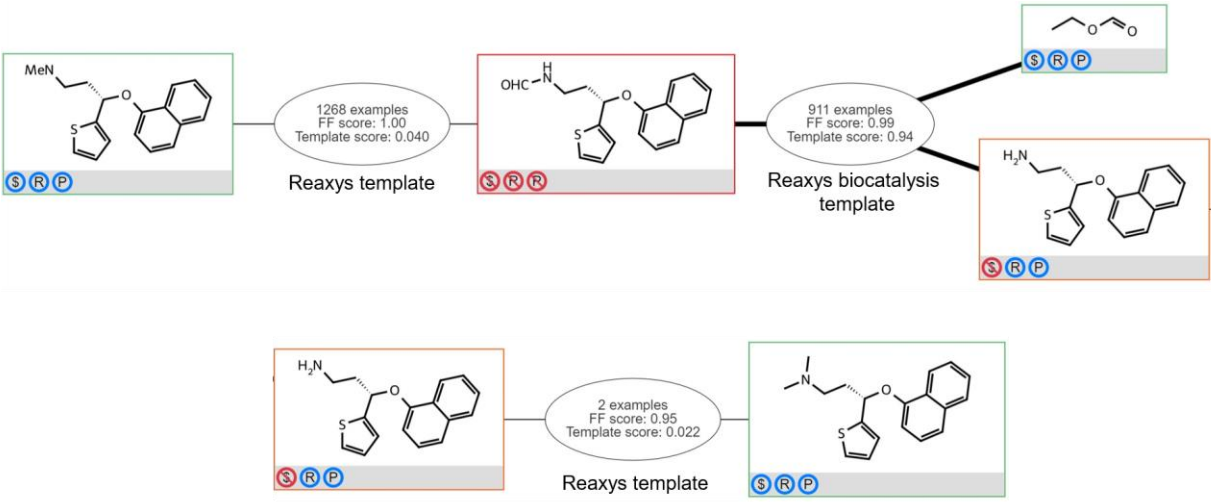

Figure S14. Top 3 scoring route of molecule **13** (first route including an enzymatic step) predicted by the ASKCOS retrosynthesis prediction tool combining the "reaxys_biocatalysis" and the "reaxys" models, other settings were left as default.

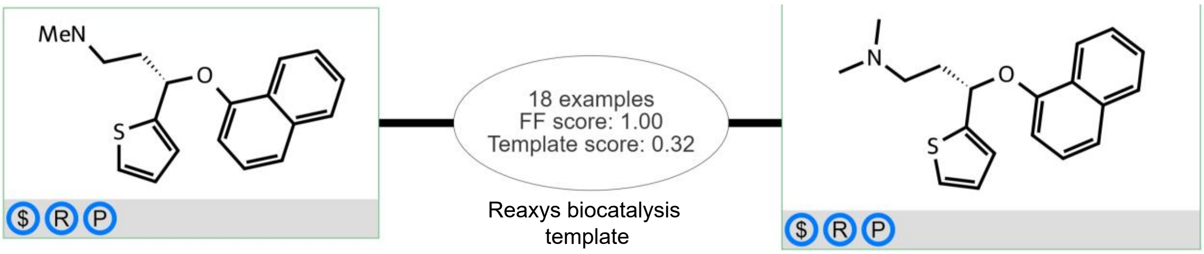

Figure S15. Single route of molecule **13** predicted by the ASKCOS retrosynthesis prediction tool using the "reaxys_biocatalysis" models only, other settings were left as default.

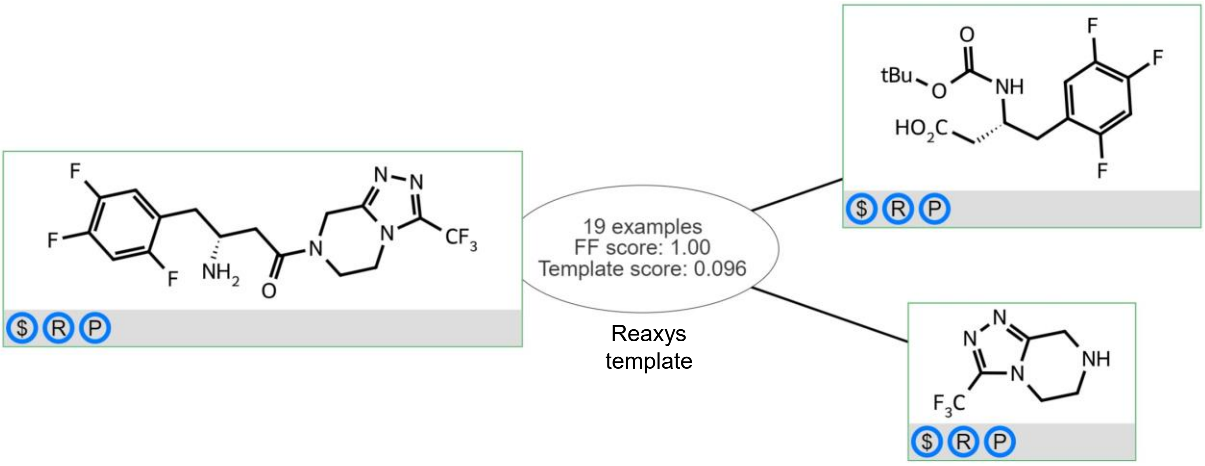

Figure S16. Top 1 scoring route of molecule **16** predicted by the ASKCOS retrosynthesis prediction tool combining the “reaxys_biocatalysis” and the “reaxys” models, other settings were left as default.

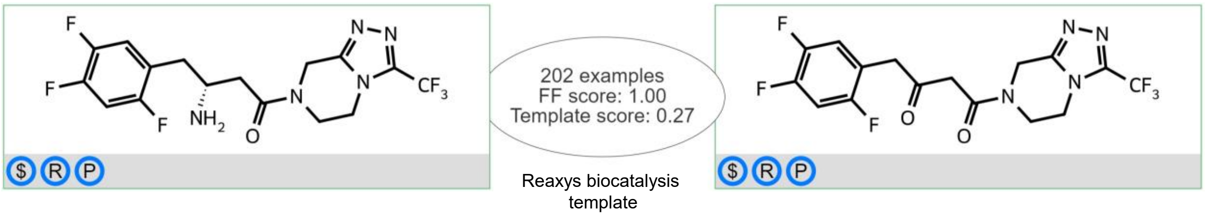

Figure S17. Top 2 scoring route of molecule **16** (first route including an enzymatic step) predicted by the ASKCOS retrosynthesis prediction tool combining the “reaxys_biocatalysis” and the “reaxys” models, other settings were left as default.

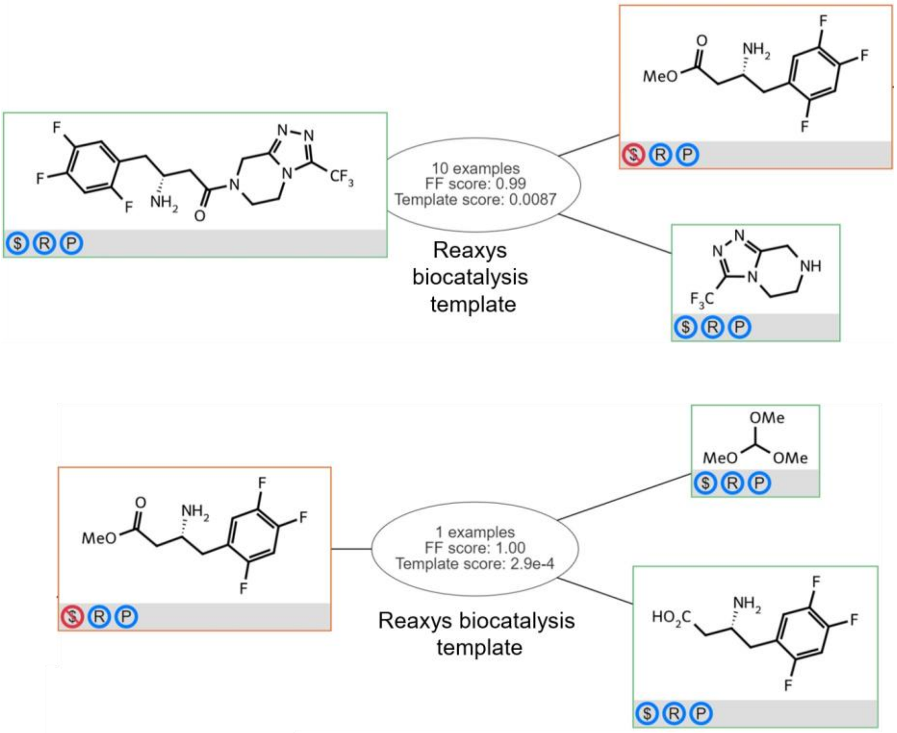

Figure S18. Top 6 scoring route of molecule **16** predicted by the ASKCOS retrosynthesis prediction tool combining the “reaxys_biocatalysis” and the “reaxys” models, other settings were left as default.

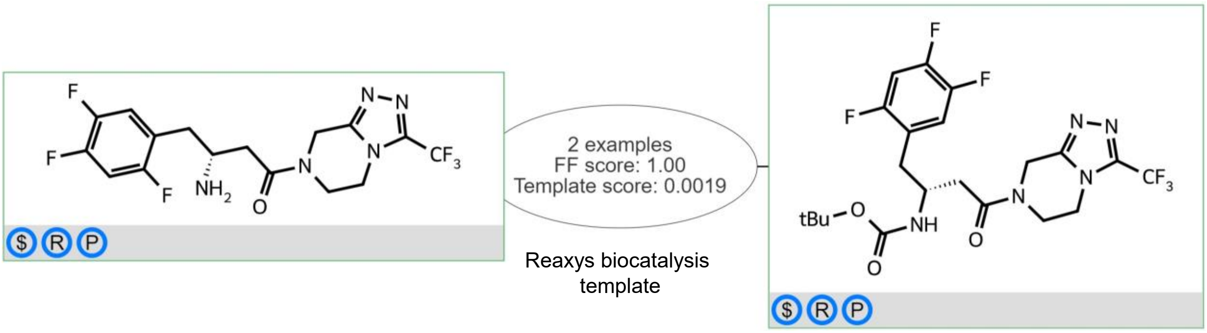

Figure S19. Top 1 scoring route of molecule **16** predicted by the ASKCOS retrosynthesis prediction tool using the “reaxys_biocatalysis” models only, other settings were left as default.

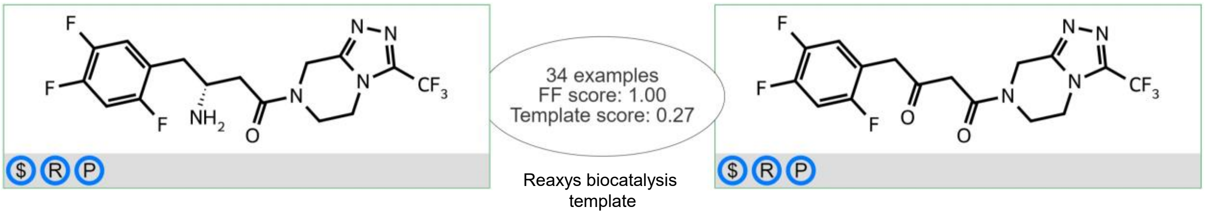

Figure S20. Top 2 scoring route of molecule **16** predicted by the ASKCOS retrosynthesis prediction tool using the “reaxys_biocatalysis” models only, other settings were left as default.

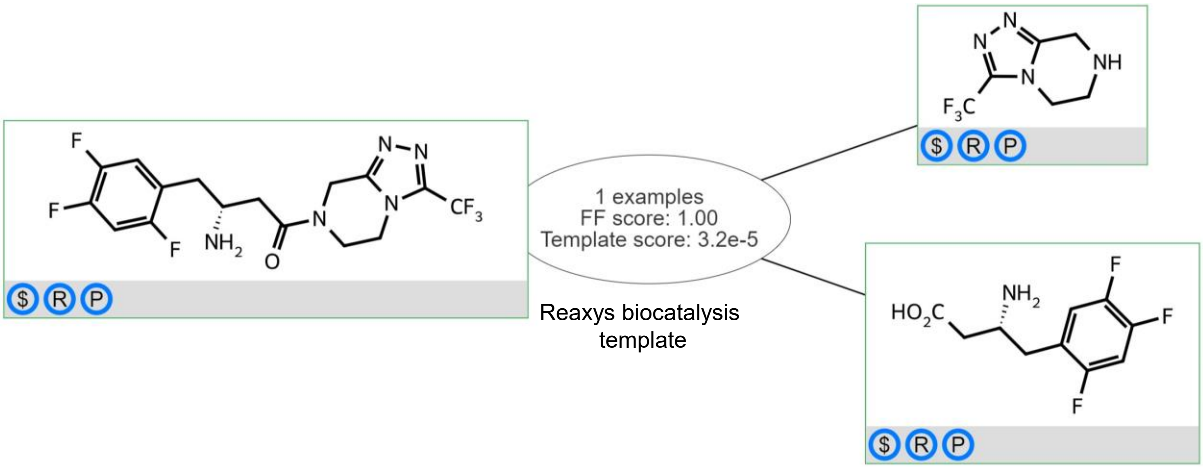

Figure S21. Top 3 scoring route of molecule **16** predicted by the ASKCOS retrosynthesis prediction tool using the “reaxys_biocatalysis” models only, other settings were left as default.

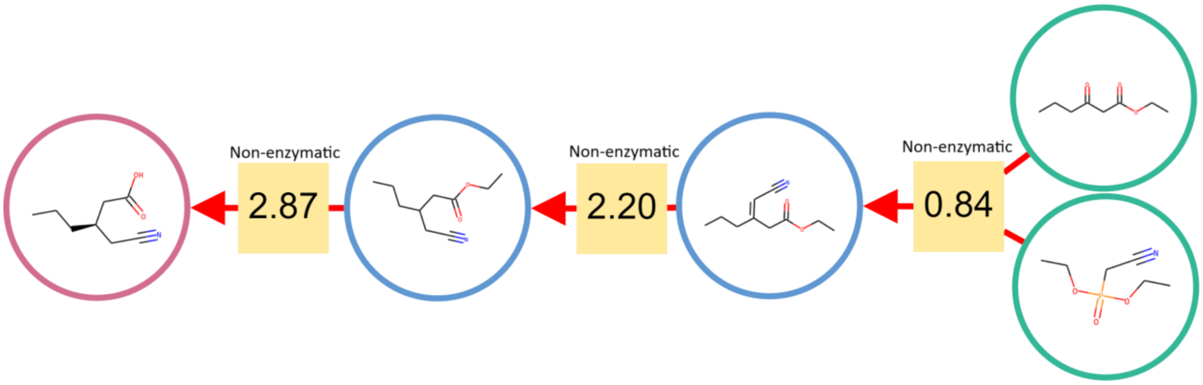

Figure S22. Top 1 scoring route of molecule **1** predicted by the BioNavi retrosynthesis prediction tool accessible at http://biopathnavi.qmclab.com/bionavi/ used with the “Default settings” preset, allowing both “Bio-building blocks” and “Chemo-building blocks”, and combining “Enzymatic synthesis” and “Non-enzymatic synthesis”.

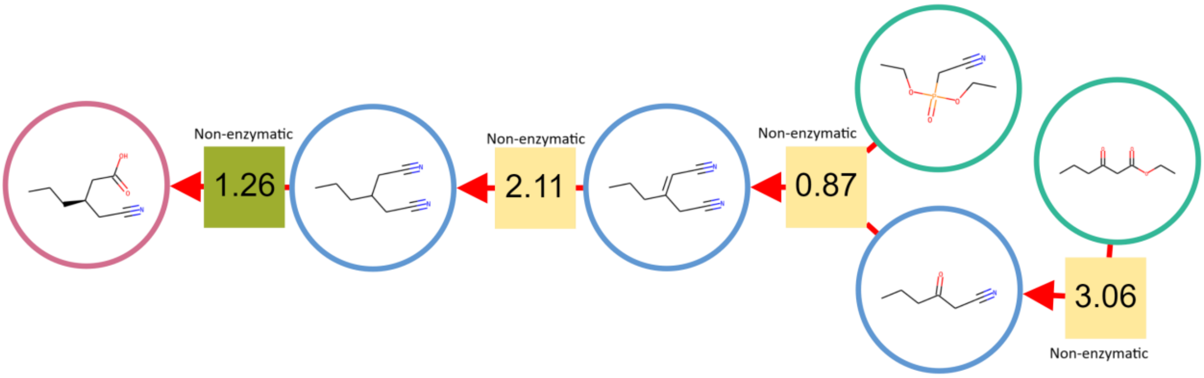

Figure S23. Top 2 scoring route of molecule **1** predicted by the BioNavi retrosynthesis prediction tool accessible at http://biopathnavi.qmclab.com/bionavi/ used with the “Default settings” preset, allowing both “Bio-building blocks” and “Chemo-building blocks”, and combining “Enzymatic synthesis” and “Non-enzymatic synthesis”.

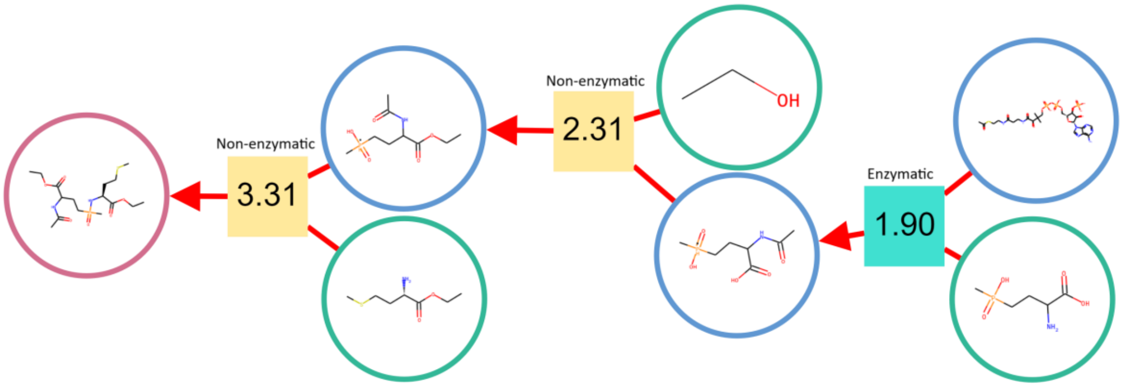

Figure S24. Top 1 scoring route of molecule **5** predicted by the BioNavi retrosynthesis prediction tool accessible at http://biopathnavi.qmclab.com/bionavi/ used with the “Default settings” preset, allowing both “Bio-building blocks” and “Chemo-building blocks”, and combining “Enzymatic synthesis” and “Non-enzymatic synthesis”.
